# Digital bridges to social connection: A systematic review and meta-analysis of digital interventions for loneliness and social isolation

**DOI:** 10.1016/j.invent.2025.100856

**Published:** 2025-07-08

**Authors:** Thomas Hansen, Rune Johansen, Benedicte Kirkøen, Kim Stene-Larsen, Melanie Straiton, Ragnhild A. Tornes, Anne Reneflot

**Affiliations:** a Department of Mental Health, Norwegian Institute of Public Health, Oslo, Norway; b Promenta Research Center, University of Oslo, Oslo, Norway; c Department of Library Services, Norwegian Institute of Public Health, Oslo, Norway

**Keywords:** Loneliness, Social isolation, Digital, Intervention, RCTs, Systematic review, Meta-analysis

## Abstract

As loneliness and social isolation emerge as pressing public health concerns, identifying effective interventions is essential. Digital solutions offer flexible, scalable, and cost-effective approaches, yet their effectiveness remains uncertain. This systematic review and meta-analysis assess the impact of randomized controlled trials (RCTs) on digital interventions to reduce loneliness and social isolation. Following PRISMA 2020 guidelines, we searched seven databases and grey literature (2022–) and applied random-effects models to pool effect sizes by intervention type. A total of 40 RCTs involving 6062 participants were included, with one-third focusing on younger individuals. Loneliness was assessed in 36 studies, while eight examined social isolation. Interventions were classified as psychological (k = 25), social (k = 4), activity-based (k = 4), robot-based (k = 4), and social media reduction (k = 5). Psychological interventions—especially those with group or social components—along with group-based activities and robotic pets, were effective in reducing loneliness. In contrast, social contact interventions, self-guided individual activities, and conversational robots showed limited impact. Social media reduction interventions suggested potential benefits, though results were not statistically significant. The evidence base exhibited moderate to high risk of bias, heterogeneity, and limited long-term follow-up. We provide specific recommendations for future interventions and research, including leveraging digital technologies for enhanced personalization, using digital tools for signposting non-digital interventions, systematically comparing digital and non-digital versions of the same intervention, and, most critically, examining the impact of increasingly popular AI-driven and humanlike social chatbots.

## Highlights

• This review evaluated RCTs on digital interventions for loneliness and social isolation
• A total of 40 RCTs with 6062 participants were included
• Psychological interventions, group-based activities, and robotic pets showed benefits
• Social support, social robots, individual activities, social media reduction showed no effect
• Evidence suggests that social and interactive components may enhance efficacy

## 1 Introduction

Social isolation and loneliness (SIL) represent distinct but interrelated aspects of social disconnection. Social isolation refers to the objective lack of relationships and interactions, while loneliness emphasizes the subjective feeling of being emotionally or socially disconnected (Cacioppo and Patrick, 2008; Hawkley et al., 2005). About 30–40 % of adults in Western nations report that they feel lonely at least some of the time, with higher rates observed among the youngest and oldest age groups (Hansen and Slagsvold, 2016; Mauri et al., 2024). Loneliness is projected to increase in absolute numbers, with rising prevalence among young people over the past two decades and a growing number of older adults affected as Western populations continue to age (Helliwell et al., 2024; Aunsmo et al., 2023). Similarly, social isolation, while varying by specific indicators (e.g., living alone, limited social network, infrequent social interaction), is especially prevalent among older age groups and has shown a general increase in recent years (Hansen et al., 2021; Kannan and Veazie, 2023). SIL is linked to a wide range of adverse physical and mental health outcomes and is increasingly recognized as critical public health concerns (Rohde et al., 2016; Hawkley and Cacioppo, 2010; Hawkley et al., 2010a; Cacioppo et al., 2006). SIL is also associated with diminished prosocial behaviors (e.g., volunteering), reduced social engagement, increased healthcare utilization, decreased productivity, and impaired daily functioning (Cacioppo and Patrick, 2008; Hawkley et al., 2010b). As a result, efforts to reduce SIL are being pursued worldwide, with governments in several countries (e.g., the Nordic countries, UK, and Japan) making loneliness a policy priority (Goldman et al., 2024).

To reduce SIL, it is essential to rely on high-quality research on intervention effectiveness. This evidence base is expanding, supported by a growing number of systematic reviews (SRs) and even umbrella reviews (URs) (Hansen et al., 2024; Lasgaard, 2022; Duffner et al., 2024). In a recent UR, we synthesized RCT-based evidence from 26 SRs on interventions targeting SIL (Hansen et al., 2024). While mixed findings limited definitive conclusions, psychological interventions—such as psychotherapy, psychoeducation, and social skills training—emerged as the most promising for addressing loneliness, as they target underlying factors such as mental health issues and maladaptive social cognitions. In contrast, social interventions (e.g., social contact and social support) had weaker and more inconsistent effects on loneliness but showed potential for reducing social isolation. However, we found that significant knowledge gaps remained, specifically regarding digital and technological interventions.

Technological advancements and the growing use of digital communication have enabled tools such as online support platforms, mobile apps, video conferencing, social chatbots, and social media to potentially mitigate SIL. The COVID-19 pandemic, with its lockdowns and physical distancing measures, further accelerated interest in these digital solutions (Welch et al., 2023; Gunnes et al., 2024). Digital interventions may offer some advantages over traditional face-to-face measures. First, digital measures can reach individuals who are unwilling to participate in interventions due to factors like stigma or shame, or unable to participate because of health issues or geographic barriers (Fritz et al., 2023). Second, digital interventions provide greater flexibility in terms of location and time, often being accessible 24/7. Third, they can be cost-effective and highly scalable, enabling outreach to larger populations (Welch et al., 2023). Finally, some of these interventions can potentially be effectively tailored to individual needs, preferences, barriers, and the specific type of loneliness experienced—critical factors for enhancing the efficacy of such measures (Hansen et al., 2024).

There is concern, however, that digital tools and communication may displace physical friendships and interactions and fail to serve as an adequate substitute, potentially increasing SIL (Askheim et al., 2024). Insofar as the human “need to belong” is best fulfilled through interactions involving eye contact, voice, gesture, touch, and smell (Baumeister and Leary, 1995), the success of digital interventions depends on their ability to foster physical social contact and relationships. Similarly, interventions may be more effective when they rely on face-to-face or group interactions, whereas solitary activities or asynchronous and indirect modes of communication—such as text messaging, social media, apps, or videos—may yield weaker effects (Fritz et al., 2023). However, the effects may vary across social groups and may be particularly beneficial for individuals who, due to factors such as old age, disability, or physical distance, are unable to engage in regular in-person interactions.

Research on digital interventions for reducing SIL remains inconclusive, as highlighted in multiple SRs (see Appendix 2 for a review of general and RCT-focused SRs). This variability stems from differences in study populations, intervention designs, measurement tools, methodologies, and study quality (Welch et al., 2023; Gunnes et al., 2024). A key weakness of this literature is the tendency to aggregate highly heterogeneous intervention types, obscuring important distinctions in their effectiveness. Additionally, reviews often fail to distinguish interventions that incorporate an interactive social element (e.g., group-based programs) from those that are purely technological (e.g., self-guided wellness apps or therapeutic chatbots). Both qualitative participant feedback and quantitative effectiveness studies suggest that group-based interaction enhances digital intervention effectiveness (Morrish et al., 2023; Nicosia et al., 2023). The evidence base is also limited, particularly in the number of high-quality RCTs—the largest SR identified only 14 RCTs (Lasgaard, 2022). However, a recent increase in such studies signals the need for an updated review (Ambagtsheer et al., 2024). Furthermore, published reviews have so far largely focused on older adults, leaving adolescents and young adults—digitally active groups with rising loneliness rates—understudied. Finally, few reviews address social isolation, despite having comparable health impacts to loneliness (Steptoe et al., 2013).

To address these gaps, we will synthesize RCT-based evidence on the effect of digital interventions for SIL, encompassing preventive and mitigating interventions across age groups. We extend prior SRs by (i) focusing exclusively on RCTs, the “gold standard” for evaluating intervention effects (Knapp, 2016), (ii) limiting the scope to recent trials (2022–) not captured in earlier SRs, (iii) including both published and grey literature, (iv) analyzing effects by intervention type and subgroup, and (v) evaluating both social isolation and loneliness. Notably, our review also aims to understand the impact of interventions targeting young adults and provide a more nuanced analysis by considering the type of intervention and the inclusion of interactive social elements, that is, interaction among participants that may foster a sense of community and belonging during the intervention. Our ultimate objective is to provide updated, evidence-based insights for researchers, policymakers, and practitioners in this field.

## 2 Methods

This systematic review and meta-analysis was registered with PROSPERO (CRD42024586950) and conducted in accordance with the Preferred Reporting Items for Systematic Reviews and Meta-Analyses (PRISMA 2020) guidelines (see Appendix 5) (Page et al., 2021).

### 2.1 Inclusion and exclusion criteria

Eligible studies were RCTs published in English in 2022 or later that focused on loneliness, social isolation, or closely related outcomes (e.g., social connectedness). All types of digital interventions were included, defined as those utilizing information and communication technology, smart devices, internet-based systems, technological tools, or robots (Balki et al., 2022). “Digital intervention” thus refers to any structured program or tool that provides support, content, or interaction primarily via digital means. All age and population groups were included. Comparators included waitlist (WL), treatment-as-usual (TAU), no treatment (NT), and alternative treatment (AT). We excluded non-digital interventions and studies that lacked SIL outcomes, studies that were non-RCTs, or where the only control group was an eligible intervention (digital treatment). Our review builds upon existing evidence syntheses, including an umbrella review covering studies published prior to 2022 (Hansen et al., 2024). As specified in our protocol, we limited inclusion to studies published from 2022 onward to capture the most recent and innovative digital solutions—reflecting current technological capabilities and social practices in a rapidly evolving digital landscape.

### 2.2 Search methods

An information specialist (RAT) conducted a systematic literature search based on a search strategy that combined text words and controlled vocabulary related to loneliness, social isolation, and digital interventions (see Appendix 1 for the complete search strategy). The strategy was peer-reviewed by a second information specialist. The search was restricted to publications from 2022 onward, and the strategy was adapted for the following databases: MEDLINE (OVID), Embase (OVID), APA PsycINFO (OVID), Sociological Abstracts (ProQuest), Web of Science Core Collection (SCI-EXPANDED, SSCI, A&HCI, and ESCI), CINAHL (EBSCO), and Cochrane Central Register of Controlled Trials. The search was performed on September 25, 2024, and updated on February 4, 2025. Search results were imported into EndNote (Gotschall, 2021), where duplicates were removed. The de-duplicated records were then uploaded into Covidence (Covidence systematic review software, 2023) for screening. The “New Cochrane RCT classifier model” AI function was used to identify potential RCTs. All references in the group “possibly an RCT” were included.

Grey literature was identified through searches in Google Scholar, Swemed+, ClinicalTrials.gov, OpenGrey, SBU (Swedish Agency for Health Technology Assessment and Assessment of Social Services), WHO, Mednar, Socialstyrelsen (The National Board of Health and Welfare, Sweden), and Statens institut for Folkesundhed (National Institute of Public Health, Denmark) on October 7–9, 2024 (updated 4 February 2025). This search covered about the same search terms and time frame as above (see Appendix 1). Including grey (unpublished) literature aimed to mitigate publication bias, which arises from the tendency to underreport non-significant findings. By incorporating such sources, the review ensures a more comprehensive and balanced assessment of available evidence.

For our literature review, a systematic search for systematic reviews was conducted on September 18, 2024, in the following databases: MEDLINE (OVID), Embase (OVID), and APA PsycINFO (OVID). The search strategy mirrored the systematic literature search conducted on September 25, with an additional methodological filter for systematic reviews (see Appendix 1).

### 2.3 Screening and selection of reviews

To ensure consistency in applying the inclusion and exclusion criteria, the research team underwent training and calibration. Screening of titles, abstracts, and full-text articles was conducted independently by two reviewers, with any disagreements resolved through discussion. Reasons for excluding full-text studies were documented (Appendix 3). For studies identified through protocols, up to two email requests were sent to the authors for copies of the completed studies. If no response was received, the study was excluded (see Appendix 3).

### 2.4 Data extraction and study quality assessment

Data extraction and risk assessment were carried out by assigning primary responsibility to one researcher, while a second researcher verified the accuracy of the extracted data. These tasks were evenly distributed among the team members. Any disagreements were resolved through consensus meetings between the two researchers, with input from the project leader (TH) if required. An Excel data extraction form was developed and piloted prior to full implementation to ensure consistency and calibration among team members.

Data were extracted on publication date, the country where the study was conducted, participant characteristics (e.g., gender, age, socioeconomic status, and recruitment method), intervention features (type, format, duration, number of sessions, and delivery mode), type of comparison group, follow-up period, numbers of participants in the intervention and control groups, and details of outcome assessments.

Based on frameworks from prior reviews and our knowledge of digital intervention research (Hansen et al., 2024), interventions were pre-classified into the following categories: social contact (facilitated peer interaction); social support (befriending or support groups); psychotherapy (targeting social cognition or distress, e.g., cognitive-behavioral therapy or mindfulness); psychoeducation (educational content related to loneliness, mental health, or well-being more broadly); social skills training (focused on improving friendship, communication, and interpersonal skills); ICT/internet training (teaching digital literacy or use of communication tools); exercise (digitally delivered physical activity programs); gaming (e.g., social or therapeutic games); telehealth (remote sessions with professionals); learning a skill/hobby (e.g., online classes in arts or languages); reduced use of social media (including abstinence interventions); mixed (interventions combining multiple categories); and other. These overlapping categories focus on the interventions' primary focus. While some interventions may include elements from multiple categories, each is assumed to emphasize one primary goal. Some of these types could be meaningfully grouped together based on conceptual similarity (Hansen et al., 2024). Specifically, social contact and social support were combined into a broader category of “social” (or direct) interventions, as they aim to directly increase social connection. Similarly, psychotherapy, psychoeducation, and social skills training were grouped as “psychological” (or indirect) interventions, targeting cognitive, emotional, and behavioral barriers to social interaction. Other categories remained separate or were excluded from group-level analysis due to conceptual heterogeneity or limited data, as discussed further below. Due to extensive calibration, the categorization process demonstrated high interrater agreement, with 89.8 % consistency between the two independent reviewers.

If the data required for calculating effect estimates were missing or unclear, the primary study authors were contacted via email, followed by a reminder if no response was received. If the authors did not reply, the study was excluded and documented in Appendix 3. Risk of bias was evaluated independently by two authors using the Cochrane Risk of Bias (RoB) Tool, version 1 (Higgins et al., 2019). The tool covers six domains of bias: selection bias (random sequence generation and allocation concealment), performance bias (blinding of participants and personnel), detection bias (blinding of outcome assessment), attrition bias (incomplete outcome data), reporting bias (selective outcome reporting), and other bias (any additional sources of bias). Each domain is rated as having “low risk,” “some concerns,” or “high risk”.

### 2.5 Data synthesis

To compare and pool effect sizes (ES), standardized mean differences (Cohen's *d*) with 95 % confidence intervals were computed using available data, as this approach accounts for variability in measurement scales across studies. ESs were calculated using post-treatment or follow-up means and standard deviations, and studies were categorized based on outcome measures and intervention type. When appropriate, we distinguish between intervention types by whether they have social/group component. ES magnitudes were classified as small (0.2), medium (0.5), and large (0.8) (Cohen, 2013). Missing standard deviations were computed using available standard errors (SE * √n) (34).

We pooled estimates using random-effect meta-analyses when at least three studies were available with similar outcomes and intervention types. When appropriate, sensitivity analyses were performed to examine variations in pooled effects between passive controls (no treatment, treatment-as-usual, or waitlist) and active controls. Heterogeneity was assessed using forest plots and the *Q* and *I*^*2*^ statistics (Higgins et al., 2019). For the *I*^*2*^ statistic, thresholds of 25 %, 50 %, and 75 %, correspond with low, moderate, and high levels of heterogeneity, respectively. For a meta-analysis with at least 10 trials included, we explored (i) heterogeneity using leave-one-out sensitivity analysis if the *I*^*2*^ value was 50 % or greater, and (ii) publication bias using funnel plots and Egger's test (Lin and Chu, 2018).

In cases of multiple relevant intervention or control arms for the same intervention type, we combined them and calculated a mean average to ensure that in each study, a single intervention group was being compared to a single control group (Cochrane Handbook section 16.5.4) (Higgins, 2013). All analysis and visualization were conducted in SPSS version 28.

## 3 Results

### 3.1 Search results and study characteristics

**Fig. 1:**
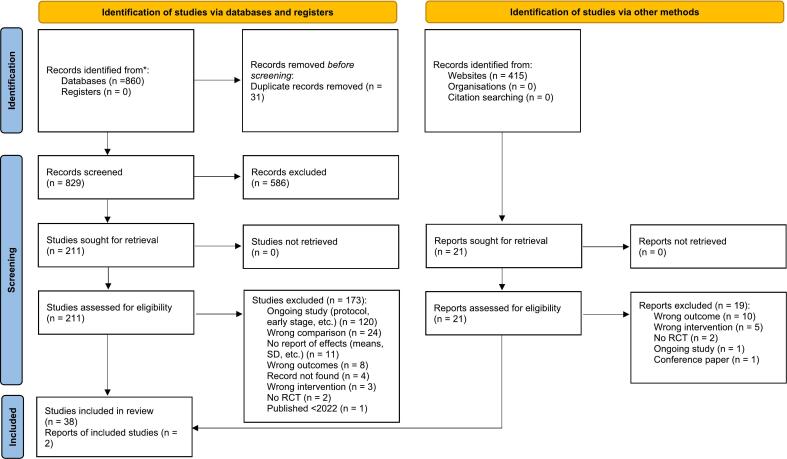
PRISMA flow chart.

The research yielded 1244 studies after duplicate removal (Fig. 1). Of these, 232 full-text articles were evaluated, and 40 studies were identified as eligible.

We included 40 studies with a total of 6062 participants (Table 1). Most studies were conducted in Western countries, with 16 from the USA, 5 from Germany, 4 from the UK, 3 from Switzerland, 2 each from the Netherlands, Poland, and Turkey, and one study each from Austria, Sweden, Portugal, South Korea, Hong Kong, and Taiwan. Regarding age (approximate classifications), 15 studies focused on younger individuals, 15 on older adults, 4 on middle-aged adults, and 6 on all age groups. Most studies (k = 35) had a clear majority (>60 %) of female participants, including two studies with only women. Based on the main strategy used, the interventions were categorized as psychological (k = 25), social (k = 4), activity-based (k = 4), robot-based (k = 4), and reduced social media use (k = 5) interventions, consolidating an initial set of 10 categories due to limited evidence in several areas.

**Table 1:** Characteristics of the included studies.

Study and country	Population (n, % women, age (SD))	Target group	Intervention vs. control^a^	# sessions, duration	Outcome and measure
Ae-Ri et al., 2023, South Korea	*n* = 40 (85 % ♀), *M*_*age*_ NR	Age 65+, community dwelling	Youtube videos, incl. Practices (therapy, mindfulness, relaxation), partly guided by research assistants vs. AT (exercise, painting)	Twice weekly, 6 weeks	Loneliness (UCLA-20)
Andrade et al., 2023, USA	*n* = 55 (43 % ♀), *M*_*age*_ = 19.3 (0.9)	Age ≥ 18, tendency to engage in social comparisons	Web/PC-delivered social savoring intervention (designed to encourage individuals to experience joy for others' positive experiences during their time on social media) vs. WL	Once, 2 weeks	Loneliness (UCLA, Social connectedness scale)
Berko et al., 2023, USA	*n* = 214 (16 % ♀), *M*_*age*_ = 60.4 (5.9)	Age 50+, HIV positive, high score on loneliness	Mindfulness (app-based audio lessons) vs. WL	14 sessions, 25 days	Loneliness (UCLA-3, daily diary)
Boucher et al., 2024, USA	*n* = 330 (67 % ♀), *M*_*age*_ = 15.2 (NR)	Age 13–17, reported elevated stress and rumination	Happify for Teens; a self-guided digital mental health program which employs evidence-based activities from CBT, positive psychology, and mindfulness to reduce perceived stress, brooding, and loneliness among adolescents vs. WL	6 sessions, 12 weeks	Loneliness (UCLA-8)
Brog et al., 2022, Switzerland	*n* = 107 (81 % ♀), *M*_*age*_ = 40.3 (NR), range 18–81	Adults 18+, presence of ≥mild depressive symptoms	ROCO, an internet-based self-help program targeting psychological distress related to COVID-19 through CBT techniques aimed at enhancing emotion regulation and resilience vs. WL	6 sessions, 3 weeks	Loneliness (UCLA-9)
Chen et al., 2024, Taiwan	*n* = 120 (IG 60.3, CG 42.3 % ♀), *M*_*age*_ = 81.8 (7.3)	Age 65+, mild dementia	Group-based program using the Personal Assistive Robot (PARO) to enhance cognitive function, autonomic nervous system regulation, and mental well-being through interactive sessions involving tactile, verbal, and group discussions with the robot vs. TAU	6 sessions, 6 weeks	Loneliness (UCLA-3)
Christie et al., 2022, Netherlands	*n* = 96 (68 % ♀), *M*_*age*_ = 56.9 (9.0), range 22–84	Primary caregivers of patients with dementia	Inlife, an online social support platform designed to enhance caregiver competence and social support vs. WL/TAU	3 sessions, 16 weeks	Loneliness (11-item DjG Loneliness scale), Social isolation (Lubben Social Network Scale-6 items)
de Hesselle and Montag, 2024, Germany	*n* = 86 (100 % ♀), *M*_*age*_ NR	University students, age 18+	Abstinence from social media vs. TAU	2 weeks	Loneliness (UCLA-3)
Demirağ and Hintistan, 2022, Turkey	*n* = 54 (50 % ♀), *M*_*age*_ = 64.1 (11.9)	Patients (age 18+) receiving hemodialysis treatment	Robot cat for 20 min during hemodialysis vs. TAU	1 session, 2 months	Loneliness (DjG scale)
DuPont et al., 2023, USA	*n* = 225 (77 % ♀), *M*_*age*_ = 18.7 (2.1)	University students	Online positive psychological intervention in which participants engage in a choice of six evidence-based activities designed to enhance positive emotions, reduce negative affect, and improve psychological well-being during the COVID-19 pandemic vs. AT (document daily activities)	7 sessions, 2 weeks	Loneliness (UCLA-8)
Dworschak et al., 2024, Switzerland	*n* = 36 (75 % ♀), *M*_*age*_ = 71.5 (4.2)	Older adults (age 65+) with high score on loneliness	Online CBT program, “NümEinsam,” designed to address loneliness by targeting maladaptive social cognition and incorporating elements of positive psychology and life-review therapy vs. WL	NR, 7 weeks	Loneliness (UCLA-9)
Faulhaber, 2023, USA	*n* = 230 (73 % ♀), *M*_*age*_ = 22.0 (5.2)	University students	Limited time on social media (30 min/day) vs. TAU	NR, 2 weeks	Loneliness (UCLA-20)
Gilbody et al., 2024, UK	*n* = 435 (62 % female) *M*_*age*_ = 75.7 (6.7)	Socially isolated, chronic health problem, and depressive symptoms	Behavioral activation program, “BASIL+,” which involves telephone sessions to help older adults address depression and loneliness by engaging in socially reinforcing activities vs. TAU (signposting to publicly available COVID-19 well-being resources)	8 sessions, 8 weeks	Loneliness (DjG scale)
Hirshberg et al., 2025, USA	*n* = 662 (87 % ♀), *M*_*age*_ NR	80 % of the sample report clinical anxiety or depression	Healthy Minds Program, a smartphone-based meditation and well-being training designed to improve mental health by enhancing mindful action, reducing loneliness, increasing cognitive defusion, and fostering a sense of purpose vs. WL	NR, 4 weeks	Loneliness (5-item NIH toolbox)
Iyer et al., 2023, USA	*n* = 108 (81 % ♀), *M*_*age*_ = 16.0 (NR), range 14–19	High school students	“Heartfulness Self-Care Program,” an online program to reduce loneliness through daily guided activities and webinars on stress management, positivity, circadian rhythm alignment, and goal setting vs. WL	NR, 4 weeks	Loneliness (UCLA-3)
Joosten et al., 2024, Netherlands	*n* = 89 (86 % ♀), *M*_*age*_ = 41.9 (7.5)	Parents of a child with cancer	“Op Koers Online,” an online group program for parents of children with cancer, designed to enhance psychosocial well-being and coping skills using CBT and acceptance and commitment therapy principles in structured chatroom sessions facilitated by trained professionals vs. WL	6 sessions, 6 weeks	Loneliness (Situation-Specific Emotional Reaction Questionnaire).
Kahlon et al., 2023, USA	*n* = 56 (80 % ♀), *M*_*age*_ = 63.0 (NR)	Depressive symptoms	Layperson-delivered, empathy-focused telephone program designed to alleviate depressive symptoms, anxiety, and loneliness through regular empathetic conversations tailored to participants' preferences for frequency and timing vs. TAU	Up to 20 sessions, 4 weeks	Loneliness (UCLA-3, DjG-6), Social isolation (Lubben Social Network Scale)
Karkosz et al., 2024, Poland	*n* = 68 (71 % ♀), *M*_*age*_ = 26.6 (NR)	Age 18–35 and subclinical depression or anxiety.	“Fido,” a Polish-language therapy chatbot delivering CBT techniques, psychoeducation, gratitude exercises, and support for cognitive distortion recognition to reduce depression, anxiety, and loneliness vs. AT (self-help book)	Unlimited, 2 weeks	Loneliness (UCLA-20)
Lippke et al., 2022, Germany	*n* = 721 (64 % ♀), *M*_*age*_ 68.3 (NR)	Older adults 60+, community dwelling	Physical activity program delivered through either web-based or print-based modes, designed to improve physical activity, social-cognitive predictors of behavior change, subjective age, and alleviate feelings of loneliness and depression in older adults vs. WL	NR, 10 weeks	Loneliness (single CES-D item)
Liu et al., 2023, Germany	*n* = 252 (73 % ♀), *M*_*age*_ = 33.9 (NR), range 18–71	Adult 18+	Animated storytelling video and written messages, designed to alleviate loneliness by promoting hope, solidarity, and coping strategies, presented either separately or in combination vs. WL	4 min	Loneliness (UCLA-8)
Maj et al., 2024, Poland	*n* = 159 (82 % ♀), *M*_*age*_ = 25.3 (NR), range 18–49	Changed residence in last 6 months, at least 18 years old. Recruitment method targeted mainly 1st year university students	Internet-based self-efficacy program, “New in Town,” consisting of eight CBT-based modules to enhance social self-efficacy, general self-efficacy, and social support among internal migrants in Poland, while also aiming to reduce loneliness and improve satisfaction with life vs. WL	8 sessions, 3 weeks	Loneliness (DjG 11-items)
Matthaeus et al., 2024, Germany	*n* = 253 (76 % ♀), *M*_*age*_ = 44.4 (NR)	Age 18–65, Berlin residency	App-delivered mental training program comparing a partner-based socio-emotional practice (Affect Dyad) and mindfulness training, focusing on daily 12-min sessions and weekly group coaching to reduce loneliness and enhance social connectedness vs. WL	60 sessions (12 min daily app-based practices and 10 2-h online coaching sessions), 10 weeks	Loneliness (UCLA-20, ecological momentary assessment)
Mueller and Cougle, 2023, USA	n = 55 (NR% ♀), *M*_*age*_ = 19.5 (2.1)	Social phobia. Most participants were students.	“Building Closer Friendships”, is a internet-based program for individuals with social anxiety disorder, incorporating emotional expressive writing, social skills practice, and exposure exercises to reduce fear of intimacy and loneliness by enhancing comfort and skill in sharing personal thoughts and emotions vs. WL	4 sessions, 4 weeks	Loneliness (UCLA-3)
Müller et al., 2024, Austria	*n* = 64 (53 % (young) and 69 % (old) ♀), *M*_*age*_ = 36.4 (16.8) and 74.4 (6.8)	Age 18+, having a relative to interact with.	Utilizing technology-supported psychoeducation to enhance intergenerational communication, reduce loneliness, and improve social connectedness among older adults and their families vs. WL	NR, 2 weeks	Loneliness (UCLA-3), Social isolation (Lubben Social Network Scale-6)
Ozturk and Tekkas-Kerman, 2022, Turkey	*n* = 61 (100 % ♀), *M*_*age*_ = 19.6 (2.3), range 18–36	Age 18+, first-year nursing student	Online laughter therapy sessions, targeting depression, anxiety, stress, and loneliness during the COVID-19 pandemic vs. TAU	8 sessions, 4 weeks	Loneliness (DjG)
Papadopoulos et al., 2022, UK and Japan	*n* = 24 (67 % ♀), *M*_*age*_ = 81.9 (9.8), range 65–98	Older adults, care home residents	Use of culturally competent socially assistive robots (“CARESSES robots”) that interact with older adults in care homes, using tailored cultural knowledge bases to enhance mental well-being, alleviate loneliness, and promote emotional connectedness through conversational engagement and activities vs. TAU	Unlimited, 2 weeks	Loneliness (UCLA-8)
Peña et al., 2024, USA	*n* = 308 (60 % ♀), *M*_*age*_ = 42.8 (NR)	Adults 18+ who report chronic pain and loneliness	Web-based game that simulates an art museum visit, where participants with chronic pain and loneliness engage in digital artwork exposure and secure attachment priming, aiming to reduce pain and social disconnection through cognitive and emotional mechanisms vs. NT	10 min	Social connectedness (12-item social disconnection scale)
Perkins et al., 2023, UK	*n* = 78 (100 % ♀), *M*_*age*_ = 35.4 (3.7)	Women with baby >9 months old. Age 26–45.	“Songs from Home,” an online songwriting program designed to reduce loneliness and postnatal depression and enhance social connectedness in women with young babies. Participants engage in weekly group songwriting workshops and asynchronous collaborative activities vs. WL	NR, 6 weeks	Loneliness (UCLA-3), Social Connectedness Revised 15-item scale
Purdie et al., 2023, USA	n = 66 (IG 89 % and CG 74 % ♀), *M*_*age*_ = NR, range 25–34	Physician in the pediatric and medicine-pediatric program at the UCLA Mattel Children's Hospital	Hybrid delivery of the Mindful Awareness Practices program, combining an initial in-person session followed by a six-week digitally delivered mindfulness meditation curriculum, designed to reduce perceived stress among pediatric resident physicians vs. WL	6 sessions, 2 weeks	Loneliness (UCLA-3)
Rubin et al., 2024, USA	*n* = 91 (60 % ♀), *M*_*age*_ = 27.3 (NR), range 18–70	Currently isolating due to COVID-19	Single-session mindfulness-based telehealth intervention aimed at reducing loneliness, perceived stress, anxiety, and depression, with an additional compassion component included in one variation to enhance emotional outcomes vs. WL	1 session (1 h)	Loneliness (UCLA-8)
Seewer et al., 2024, Switzerland	*n* = 243 (79 % ♀), *M*_*age*_ = 45.8 (NR), range 19–80	Reporting to be lonely.	Online CBT and mindfulness. IG1 received personal feedback from coach, IG2 only standardized written motivational messages vs. WL	9 sessions, 9 weeks	Loneliness (UCLA-9), Social isolation (Social Network Index)
Silva et al., 2024, Portugal	*n* = 70 (73 % ♀), *M*_*age*_ = 71.6 (IG) and 67.9 (CG) (NR)	Older adults 60+, living independently, no cognitive impairment	“DanceMove,” a web-based digital solution delivering an unsupervised, step-based physical activity program for eight weeks, designed to improve physical, cognitive, and psychosocial functioning in community-dwelling older adults vs. NT	Recommended 20–30 min, 3 times per week, 8 weeks	Loneliness (UCLA-6)
Teo et al., 2025, USA	*n* = 52 (38.6 % ♀), *M*_*age*_ = 67.6 (5.9)	Rural older adults 60+	Telehealth yoga classes delivered via video conferencing in a group format, incorporating a socializing component before and after each session, though the primary focus remains on yoga practice vs. WL	8 sessions, 10 weeks	Loneliness (UCLA-20, NIH-5), Social isolation (DSSI-Social Interaction)
Tunçgenç et al., 2024, UK	*n* = 58 (90 % ♀), *M*_*age*_ = 13.4 (1.6)	Adolescents aged 11–16	Online group dance program focusing on hip-hop choreography, designed to enhance social bonding, well-being, and future orientation among adolescents during the COVID-19 pandemic vs. WL	5 sessions, 5 weeks	Social bonding (4 items)
Wagner et al., 2024, Germany	n = 107 (IG 62 % ♀, CG 73 % ♀), *M*_*age*_ = 17.2 (2.1)	Adolescents aged 14–21 with care experience (foster care, institutions, or adoption).	EMPOWER YOUTH: a six-module, internet-based prevention program focusing on enhancing emotion regulation, self-appraisal, and risk recognition to help youth with care experience develop strategies to prevent victimization and cope with its psychological effects vs. WL	6 modules (45 min. each), 12 weeks	Loneliness (SOEP 3-items)
Walsh et al., 2023, USA	*n* = 912 (68 % ♀), *M*_*age*_ = 19.4 (5.2)	University students	Two gratitude expression activities—texting gratitude to benefactors (IG1) or sharing gratitude publicly on social media (IG2) conducted to enhance well-being, reduce loneliness, and foster social connectedness among undergraduate students vs. AT (document daily activities)	NR, 1 week	Loneliness (CIT, 3-items), Social connectedness (BMPN, 3-items)
Walsh et al., 2024, USA	*n* = 338 (78.1 % ♀), *M*_*age*_ = 19.4 (2.4)	Undergraduate students	A Digital Diet group, which restricted digital media use (e.g., gaming, news, and entertainment apps) (IG1), and a Social Diet group, which restricted social media use (e.g., Facebook, Instagram, Snapchat) (IG2) vs. active control (Water Diet) group, which restricted water use (e.g., reducing water consumption during daily activities) (CG1) and NT (CG2)	NR, 8 days	Loneliness (MtF loneliness scale, 6-items), social connectedness (BMPN, 3-items)
Warner et al., 2024, Hong Kong	*n* = 375 (78 % ♀), *M*_*age*_ = 63.5 (4.8)	Age 50–70, reporting loneliness	Training older adults to serve as telephone-based lay counselors, delivering psychosocial interventions such as behavioral activation, mindfulness, and befriending to reduce loneliness and improve mental health outcomes among their peers vs. AT	2 sessions per week, 6 months	Loneliness (UCLA-20, DjG-6), Social isolation (Lubben Social Network Scale)
Wolgast et al., 2023, Sweden	*n* = 174 (76 % ♀), *M*_*age*_ = 22.3 (2.9)	University student age 18–29	Reduced use of social media. Reducing social networking site (SNS) usage to 30 min daily (IG1) or shifting to passive SNS use for three weeks (IG2), aimed at improving psychological well-being, including reducing loneliness, stress, and depression vs. NT	NR, 3 weeks	Loneliness (UCLA-3)
Xiang et al., 2024, USA	n = 64 (81 % ♀), *M*_*age*_ = 69.8 (NR), range 60–86	Age 60+, depressive symptoms	Internet-based CBT program, “Empower@Home,” tailored for older adults, combining web-based self-help modules and weekly phone coaching sessions by trained laypersons to reduce depression, anxiety, and social isolation vs. WL	9 sessions, 10 weeks	Social isolation (PROMIS, 8-items)

a AT = Alternative treatment, TAU = Treatment as usual, NT = No treatment, WL = Waitlist.

The most frequently studied outcome was loneliness (k = 36), followed by social isolation (k = 8), and social connectedness (k = 4). Usually, these outcomes were among the studies' primary outcome (k = 32); however, in 8 studies these were secondary outcomes and the primary emphasis was on issues such as symptoms of anxiety or depression (Brog et al., 2022; Boucher et al., 2024; Gilbody et al., 2024; Joosten et al., 2024; Tunçgenç et al., 2024; Maj et al., 2024; Silva et al., 2024; Xiang et al., 2024). Loneliness was most often measured using versions (e.g., 3-item and 20-item) of the UCLA loneliness scale (k = 27) or other scales such as the De Jong Gierveld Loneliness Scale (k = 5). Social isolation was primarily measured with the Lubben Social Network Scale (k = 4), and social connectedness with the Social Connectedness Scale (k = 3).

Aside from singular sessions (the shortest lasted 4 min), the duration of interventions varied from one week to 24 weeks, in which session frequencies ranged from daily engagement to once a week. Twenty-two studies used a waitlist control, 13 used treatment-as-usual or no treatment, and 6 employed alternative activity as controls, yielding 41 control groups across 40 studies (due to one study having two controls).

The evidence base was characterized by moderate to high risk of bias (see Appendix 4), with substantial heterogeneity and uncertainty in effect estimates. Most studies described procedures for randomization and allocation of participants, and few selectively reported outcomes. However, significant concerns arose from the lack of blinding of participants to interventions, as well as incomplete outcome data and non-blinded outcome assessments in about half the studies. In terms of overall assessments, two studies were assessed as having low risk of bias, 17 had unclear risk, and 21 had high risk of bias.

In the findings presented below, we primarily focus on loneliness outcomes, unless stated otherwise. We also provide brief comments on key study characteristics, including the comparator and whether the studied population was “at-risk”—experiencing SIL or associated risk factors, such as mental health issues. Of the 10 studies reporting follow-up data, five studies reported on follow-up assessments 2–4 weeks, and 5 studies 2–4 months following post-test. Forrest plots with two or fewer studies can be found in Appendix 6.

### 3.2 Psychological interventions

We included 25 psychological interventions designed to enhance positive emotions or reduce stress and cognitive biases that may obstruct meaningful interpersonal relationships. These interventions used cognitive-behavioral therapy (CBT), stress management, positive psychology lessons and practices (e.g., gratitude exercises, optimism training, acts of kindness, and relationship-building activities), mindfulness, or a combination. Notably, seven psychological interventions explicitly targeted loneliness or distress during the COVID-19 pandemic (Brog et al., 2022; Gilbody et al., 2024; Tunçgenç et al., 2024; DuPont et al., 2023; Ozturk and Tekkas-Kerman, 2022; Teo et al., 2025; Rubin et al., 2024). We separated studies that were group-based versus individual and self-guided. Of the latter we grouped mindfulness as a separate category.

**Fig. 2:**
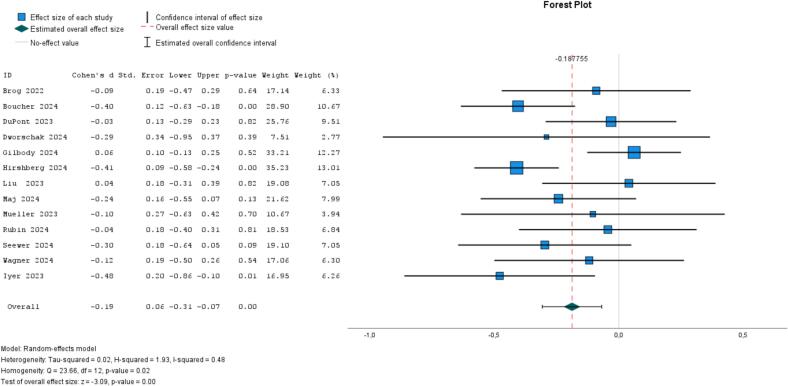
Meta-analysis of self-guided psychological interventions on loneliness (13 studies).

**Fig. 3:**
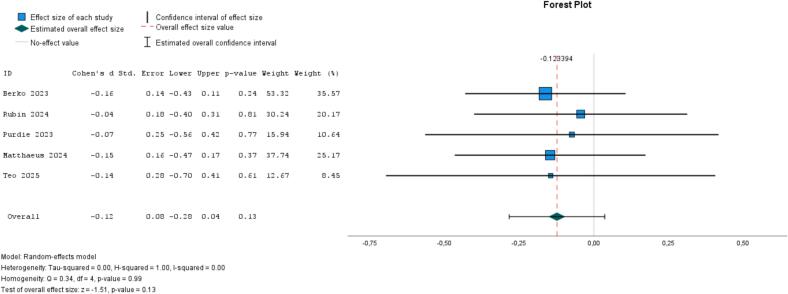
Meta-analysis of self-guided mindfulness/yoga on loneliness (5 studies).

A total of 13 studies (Brog et al., 2022; Boucher et al., 2024; Gilbody et al., 2024; Maj et al., 2024; DuPont et al., 2023; Rubin et al., 2024; Seewer et al., 2024; Dworschak et al., 2024; Wagner et al., 2024; Hirshberg et al., 2025; Iyer et al., 2023; Liu et al., 2023; Mueller and Cougle, 2023) examined self-guided, individual-based psychological interventions (with loneliness as the outcome), with half targeting at-risk populations. All but one study (DuPont et al., 2023) employed a waitlist or no-treatment control group. The funnel plot showed a symmetrical distribution of effect sizes, suggesting no evidence of publication bias (see supplementary analyses in Appendix 6). The pooled ES showed evidence of a small effect (−0.19, 95 % CI, −0.31 to −0.07, *I*^*2*^ = 48 %, *P* = .02; Fig. 2). When we exclude an outlier (Gilbody et al., 2024), the ES was −0.23 (95 % CI, −0.34 to −0.11, *I*^*2*^ = 33 %, *P* = .20). When limiting the analysis to studies that identified loneliness as their primary outcome, we found a nearly identical pooled effect size to the full model including all 13 studies (−0.20, 95 % CI, −0.35 to −0.06, *I*^*2*^ = 38 %, *P* = .01; Appendix 6). When comparing age groups, the six studies involving younger participants yielded more evidence of effect (ES = −0.24, 95 % CI, −0.40 to −0.08, *I*^*2*^ = 30 %, *P* = .00) compared to the six studies with older participants (ES = −0.12, 95 % CI, −0.29 to 0.05, *I*^*2*^ = 56 %, *P* = .18) (Appendix 6). Some studies explored whether the presence of (digital) professional support influenced outcomes, with findings suggesting a trend—though not statistically significant—toward greater benefits when professional support was provided compared to fully self-guided interventions (Gilbody et al., 2024; Seewer et al., 2024). A pooled analysis of five studies of mindfulness (vs. waitlist) among university students found no evidence of effect (ES = −0.12, 95 % CI, −0.28 to 0.04, *I*^*2*^ = 0 %, *P* = .99; Fig. 3) (Teo et al., 2025; Rubin et al., 2024; Berko et al., 2023; Matthaeus et al., 2024; Purdie et al., 2023).

**Fig. 4:**
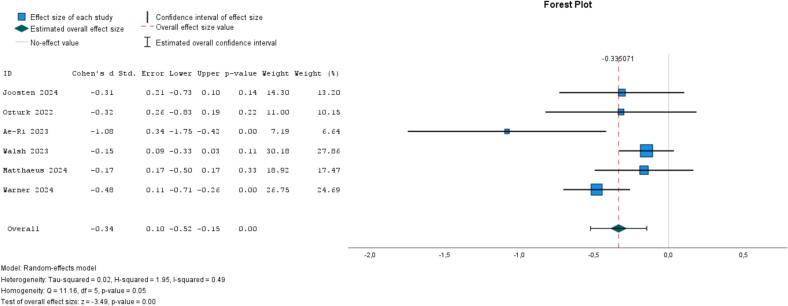
Meta-analysis of group-based psychological interventions on loneliness (6 studies).

Six studies compared group-based psychological interventions, compared with alternative treatment (k = 3), waitlist (k = 2), or treatment-as-usual (k = 1), reporting a small-to-moderate effect (ES = −0.34, 95 % CI, −0.52 to −0.15, *I*^*2*^ = 49 %, *P* = .05; Fig. 4) (Joosten et al., 2024; Ozturk and Tekkas-Kerman, 2022; Matthaeus et al., 2024; Ae-Ri et al., 2023; Walsh et al., 2023; Warner et al., 2024). In contrast to above, the two studies of younger individuals showed an ES of −0.17 (95 % CI, −0.34 to 0.00, *I*^*2*^ = 53 %, *P* = .06), against −0.43 (95 % CI, −0.69 to −0.17, *I*^*2*^ = 50 %, *P* = .08) in the four studies of older individuals (Appendix 6).

A total of five studies explored social isolation or social connectedness outcomes. Among self-guided, individual-based interventions, two studies assessed social isolation in at-risk populations and showed no effect (ES = −0.16 (95 % CI, −0.44 to 0.13, *I*^*2*^ = 0 %, *P* = .28) (Xiang et al., 2024; Seewer et al., 2024). One study on social connectedness also found no effect (Peña et al., 2024). In contrast, among group-based interventions, one study on social isolation (Warner et al., 2024) and one study on social connectedness (Walsh et al., 2023) both reported significant effects. One study assessed the effect of mindfulness on social isolation, finding no effect (Teo et al., 2025).

Follow-up data on loneliness was available for five studies. Among these, three individual-based studies reported outcomes 1–2 months post-test, and showed evidence of effect (ES = −0.22, 95 % CI: −0.41 to −0.03; *I*^*2*^ = 45 %, *P* = .21) (Gilbody et al., 2024; Hirshberg et al., 2025; Mueller and Cougle, 2023). Additionally, one mindfulness study assessed at 2 weeks (Joosten et al., 2024) and one group-based study assessed at 4 months post-test (Berko et al., 2023) found no evidence of effect.

### 3.3 Social interventions

**Fig. 5:**
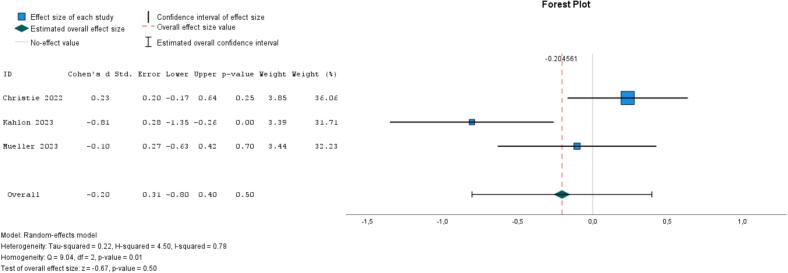
Meta-analysis of social interventions on loneliness (3 studies).

**Fig. 6:**
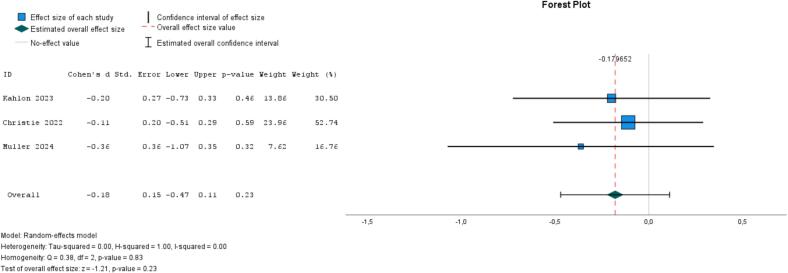
Meta-analysis of social interventions on social isolation (3 studies).

Two studies compared social support interventions with treatment-as-usual for at-risk populations; one evaluated an online platform for caregivers of individuals with dementia, while the other examined the effects of telephone-based volunteer support for individuals with depressive symptoms (Christie et al., 2022; Kahlon et al., 2023). Additionally, a social contact intervention (vs. waitlist) utilized digital communication tools to enhance intergenerational communication and social connectedness among older adults and their families (Müller et al., 2024). There was no strong evidence of effect, as the pooled ES for loneliness was −0.20 (95 % CI, −0.80 to 0.40, *I*^*2*^ = 78 %, *P* = .50) and for social isolation −0.18 (95 % CI, −0.47 to 0.11, *I*^*2*^ = 0 %, *P* = .83) (Fig. 5, Fig. 6). When removing the social contact study and isolating the effect of social support, the ESs were, respectively, −0.27 (95 % CI, −1.29 to 0.75, *I*^*2*^ = 89 %, *P* = .60) and − 0.14 (95 % CI, −0.46 to 0.18, *I*^*2*^ = 0 %, *P* = .79).

### 3.4 Robot-based support

**Fig. 7:**
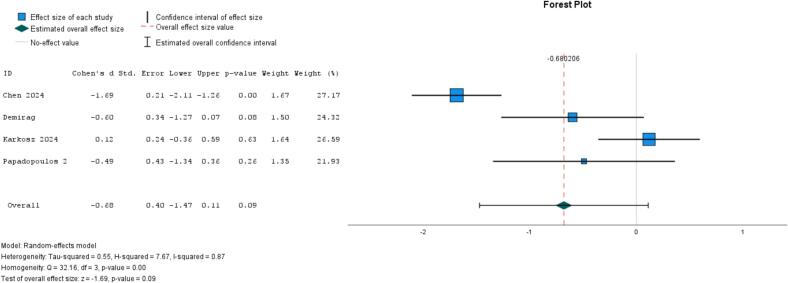
Meta-analysis of robot-based interventions on loneliness (4 studies).

Four studies evaluated the effectiveness of robotics interventions (vs. no treatment) in at-risk populations: two on robotic pets (robotic cat, robotic seal) among older people in long-term care settings (Chen et al., 2024; Demirağ and Hintistan, 2022) and two on chatbot-based conversational robots in long-term care (Papadopoulos et al., 2022) or among younger individuals with mental health challenges (Karkosz et al., 2024). While the overall effect was not significant (ES = −0.68, 95 % CI, −1.47 to 0.11) and showed high heterogeneity (*I*^*2*^ = 87 %, *P* = .00) (Fig. 7), there was evidence of an effect for robotics pets (ES = −1.17, 95 % CI, −2.24 to −0.11, *I*^*2*^ = 86 %, *P* = .01), but not for conversational robots (ES = −0.08, 95 % CI, −0.64 to 0.48, *I*^*2*^ = 33 %, *P* = .22). Similarly, follow-up data at 4 months showed an effect for robotics pets (Chen et al., 2024), but not for conversational robot (Karkosz et al., 2024).

### 3.5 Activity-based

Four studies compared activity-based interventions with waitlist or no treatment, including two group-based (Tunçgenç et al., 2024; Perkins et al., 2023) and two individual-based (Silva et al., 2024; Lippke et al., 2022) activities, compared with waitlist or no treatment. The group-based ones involved online songwriting (for new mothers) and dance (for girls aged 11–16). The self-guided, individual studies evaluated online weekly advice on physical activity (for a group, yet with minimal interaction among participants) and an online dance program. The individual studies showed no effect (ES = −0.09, 95 % CI, −0.50 to 0.32, *I*^*2*^ = 60 %, *P* = .11), whereas the group-based interventions demonstrated an effect on social connectedness (ES = 1.07, 95 % CI, −0.32 to 2.46, *I*^*2*^ = 93 %, *P* = .00). Only one group-based study assessed loneliness, reporting a significant effect (Perkins et al., 2023).

Two studies provided follow-up data, one on individual-based activities (three months post-test) (Silva et al., 2024) and one on group-based activities (one month post-test) (Perkins et al., 2023), with both showing no evidence of effect.

### 3.6 Reduced use of social media

**Fig. 8:**
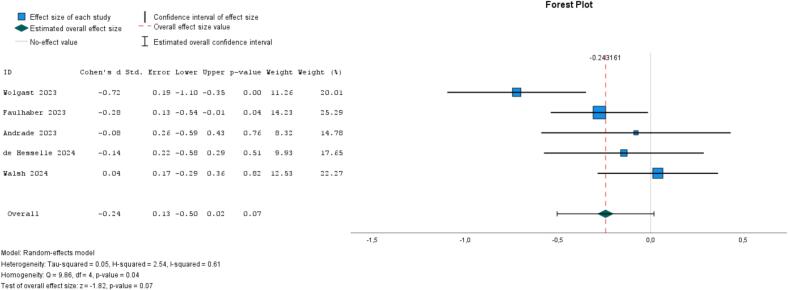
Meta-analysis of reduced use of social media on loneliness (5 studies).

Five studies assessed reduced use of social media (vs. no treatment) among young people (Andrade et al., 2023; Walsh et al., 2024; Wolgast et al., 2023; de Hesselle and Montag, 2024; Faulhaber, 2023), showing a trend toward a small effect but no strong evidence (ES = −0.24, 95 % CI, −0.50 to 0.02, *I*^*2*^ = 61 %, *P* = .04; Fig. 8). Two studies found no effect on social connectedness (ES = 0.05, 95 % CI, −0.31 to 0.41, *I*^*2*^ = 35 %, *P* = .22) (Andrade et al., 2023; Walsh et al., 2024). One study with a three-month follow-up also found no effect (de Hesselle and Montag, 2024).

## 4 Discussion

Given the rapid advancement of digital interventions for SIL, reviewing recent evidence is particularly valuable. Driven by the growing adoption of digital communication technologies—especially during the COVID-19 pandemic—this meta-analysis focused on studies published from 2022 onward and included 40 RCTs with over 6000 participants, roughly twice the number captured in previous reviews of pre-2022 studies (Hansen et al., 2024; Lasgaard, 2022). Nearly half of interventions were selective, targeting individuals reporting loneliness, social isolation, or known risk factors (e.g., health issues), the rest being universal. Most studies originated from the USA and Northwestern Europe, with women overrepresented in nearly all samples. Interventions were categorized into psychological, social, robot-based support, activity-based, and social media reduction approaches, with distinctions between individual and group-based formats where applicable. Overall, digital interventions had variable and sometimes uncertain or not significant effects. The most pronounced effects were observed for psychological approaches—especially those with a social component—along with group-based activities and robotic pets. Follow-up data were limited but suggested that most interventions had weak or no long-term effects. The evidence base had a moderate to high risk of bias, primarily due to issues with blinding and incomplete or unclear reporting of methodological procedures.

Psychological interventions were the most common, ranging from CBT to positive psychology exercises and mindfulness, aiming to reduce negative thought patterns and emotions that may hinder social connection. Often targeting at-risk groups, these interventions showed small-to-moderate benefits, particularly when including a social or interactive component. Findings suggest that interactive, in-person elements may be key, though further research is needed. Notably, our effect sizes (*d* = −0.16 for individual and − 0.34 for group-based approaches) were slightly lower than those reported in systematic reviews of non-digital psychological interventions (*d* ≈ −0.50) (Hansen et al., 2024; Lasgaard, 2022), suggesting that digital formats may be less effective in reducing loneliness. While prior studies highlight the role of social interaction in reducing loneliness, the absence of direct comparisons between online and offline interventions leaves uncertainty about their relative effectiveness—an important consideration for policy and practice. Another area for research concerns the added benefit of human, professional support. While our review found a non-significant trend toward greater benefits in interventions that included (digital) professional support compared to fully self-guided formats (Gilbody et al., 2024; Seewer et al., 2024), this aligns with earlier evidence suggesting that internet-delivered CBT with professional guidance can yield moderate reductions in loneliness in pre-2022 studies (Käll et al., 2020). Follow-up data were available for three studies on individual, self-guided interventions, showing similar effects at post-test and at 1–2 months follow-up, suggesting a degree of sustained impact for these approaches.

Social interventions, such as online meeting places and social support, had highly variable effects, though evidence is limited to three RCTs. Social support, including volunteer companions and support groups, yielded no evidence of effect on either social isolation or loneliness. Similarly, an intervention promoting intergenerational contact among family members found no effect on either outcome. These findings align with research on offline interventions, where social meeting places and support interventions often show inconsistent effects on loneliness (Hansen et al., 2024). Similarly, systematic reviews of RCTs on digital social networking platforms (Balki et al., 2022) and web-based group discussions (Shah et al., 2021) found no evidence of impact on loneliness among older adults. The uncertainty surrounding social interventions suggests they may not alleviate loneliness unless they also address underlying psychological factors such as mistrust, low self-esteem, fear of rejection, and social anxiety (Masi et al., 2011; Qualter et al., 2013). These insights suggest that fostering social connections within a safe environment may be ineffective unless also targeting the psychological roots of loneliness (Käll et al., 2020). Moreover, it may seem contradictory that social interventions lack strong evidence of effect, while psychological-behavioral interventions seem especially effective when they incorporate a social element. If replicated, the discrepancy may stem from the reciprocal nature of interaction in the latter. While genuine social connectivity relies on reciprocity—both giving and receiving support (Office of the Surgeon General, 2023)—social support interventions often involve primarily one-way support, which may limit their impact. Additionally, group-based interventions may foster more enduring social connections beyond the intervention period. In contrast, the abrupt termination of befriending interventions can lead to a “return to normal” or even a sense of loss, potentially resulting in stable or even increased loneliness at post-test.

Robot-based companionship and support had variable effects, with robotic pets for older adults demonstrating strong effects on loneliness, whereas conversational robots had minimal impact. These findings align with a previous pooled analysis of four similar RCTs—combining robotic pets and conversational robots in nursing homes—which reported a small-to-moderate but non-significant effect on loneliness (Sha et al., 2024). Our findings suggest that robotic pets can be particularly beneficial. Beyond reducing loneliness, robotic pets in long-term care have also been linked to broader benefits, including improved mental health and overall well-being (Koh et al., 2021). Conversational robots may hold more promise—and challenges—as future social chatbots powered by large language models evolve, offering increasingly tailored and humanlike social interaction and support (see below).

We identified four studies of activity-based interventions: two group-based and two individual interventions. Group-based interventions, such as online songwriting and dance, showed positive effects on loneliness. In contrast, individual interventions, such as dance and physical activity education programs, demonstrated no effects. Findings reinforce the importance of social and interactive elements in achieving meaningful outcomes. Activity-based interventions for loneliness have been widely tested in non-digital formats, with weak and variable effects (Hansen et al., 2024). Two systematic reviews have examined the effects of gaming, often combined with physical exercise, on loneliness among older adults. The findings from these reviews are inconclusive (Forsman et al., 2018; Li et al., 2018), yet they lack a distinction between individual and group-based activities.

Although based on a limited number of studies, findings suggest that group-based activity interventions may be more effective in reducing loneliness than individual-focused approaches. This aligns with research showing that people generally prefer group-based formats with shared interests over solitary interventions (Morrish et al., 2023; Nicosia et al., 2023; Teo et al., 2025). Such interventions promote active participation, mutual engagement, and collective experiences, fostering a sense of fellowship and belonging.

Interventions aimed at reducing social media use among students showed a trend toward positive effects, though results were not statistically significant. This aligns with previous research indicating a complex relationship between social media use and loneliness, where excessive engagement is linked to increased loneliness, particularly among younger individuals (d'Hombres and Gentile, 2024). It also reflects findings from an earlier SR, which reported that reducing social media use was effective in two out of five RCTs (Plackett et al., 2023). These modest effects may reflect that the outcomes depend on several factors. For example, the impact can vary based on individuals' typical style of engagement (active versus passive use), their level of offline social involvement, and whether the reduction in usage is adopted collectively by their social network. If an individual reduces their usage while peers continue high engagement, this mismatch could inadvertently lead to a heightened sense of isolation and loneliness.

### 4.1 Strengths, limitations, and future directions

Strengths of this review include the use of rigorous methods and quality assessment, grouping studies by type of intervention and outcome, the exclusive focus on RCTs, and the broad search strategy including grey literature. Notably, this is the first review to incorporate a significant number of studies on young people, addressing a critical gap given the high rates of loneliness in this population.

However, several limitations should be noted. Intervention categorization relied on face validity, which may introduce subjectivity. Another limitation relates to the conceptual boundaries of what constitutes a digital intervention. Some included interventions—such as telephone-based programs and robotic pets—do not align neatly with conventional notions of digital interventions, though they meet key criteria like scalability and independence of location. These interventions might more accurately be described as technology-assisted rather than strictly digital, and future research should work toward clearer definitions to guide inclusion criteria and interpretation of findings in this evolving field. Additionally, heterogeneous interventions in intervention design, populations, and comparator were combined in meta-analyses to enhance statistical power, potentially influencing effect estimates and obscuring the effect of more specific comparisons. Similarly, the sustainability of effects, publication bias, and sources of heterogeneity could not be fully explored due to the limited number of studies per meta-analysis. Furthermore, long-term follow-up data is scarce, limiting insights into sustained effects. Most studies relied on passive controls, which may inflate effect sizes due to the absence of an active comparator that accounts for general engagement effects. Many trials lack clear theoretical bases, making it difficult to identify key mechanisms and understand how interventions (e.g., mindfulness and other self-guided approaches) are expected to impact SIL. Notably, when we exclude studies where SIL was not the primary outcome, the pooled effect sizes by intervention type remain virtually unchanged, suggesting robustness of our findings. We suggest that future research prioritize interventions with a clear theoretical grounding and explicitly target SIL as a primary outcome to enhance both interpretability and relevance.

Furthermore, many studies were conducted during COVID-19, raising concerns about generalizability and highlighting the need for replication in a post-COVID context (Morrish et al., 2023). Also, although we review and integrate earlier findings, our findings are based on evidence from 2022 onwards, as per our protocol. This because we wanted to focus on current practices and tools, in a rapidly changing field. We realize, however, that more robust and powered meta-analysis would be possible with a more inclusive search and analysis (including also earlier studies).

Key gaps in the literature remain, including a lack of research on social isolation—despite its comparable health impacts to loneliness—as well as on male participants and subgroup differences (e.g., gender and age) in intervention effects. Future research should directly compare individual vs. group interventions and digital vs. non-digital formats to identify their relative advantages. Similarly, whether interventions specifically target lonely individuals or those at risk remains unclear, highlighting the need for greater clarity in future intervention design and evaluation. Future research should also aim to increase sample size and improve transparency in reporting by clearly detailing the implementation of key methodological procedures to reduce the risk of bias (Hansen et al., 2024).

Finally, several technologies with significant implications for SIL remain largely unexplored. For instance, while concerns exist, online multiplayer gaming among youth and young adults may foster meaningful social connections, as suggested by our findings regarding the benefits of interactive elements. Additionally, no RCT-based studies have examined AI-driven social chatbots (e.g., Replika), which use large language models to generate personalized, open-ended conversations often indistinguishable from human speech. Future iterations may integrate voice and embodied features (e.g., humanoid or animal-like robots) for more lifelike interactions. Early data suggest users form supportive, non-judgmental relationships with these agents (Skjuve et al., 2021; Xie and Pentina, 2022). Despite risks such as addiction or withdrawal from human interaction, AI could offer a safe space for self-expression and connection, particularly for vulnerable individuals (Allen et al., 2024).

Future interventions should also leverage digital tools to tailor interventions to specific types and causes of loneliness, which is key to enhancing their effectiveness (Morrish et al., 2023; Barbosa Neves et al., 2023). Unlike offline approaches, they can better adapt to individual needs, preferences, and different types of loneliness and triggers, moving beyond a one-size-fits-all approach (Barbosa Neves et al., 2023). Similarly, these tools can also facilitate combining elements from various approaches—such as therapeutic counseling and social interaction—which may offer a more holistic, tailored, and effective solution (Hansen et al., 2024). Relatedly, future research should explore how digital platforms can facilitate real-world, in-person connections. This could involve digital signposting interventions, directing individuals to community services and social activities tailored to their interests and needs, similar to social prescribing models. These online platforms already exist in many countries to connect individuals with local events, activities, and social opportunities (e.g., www.genlyd.dk).

## 5 Conclusion

This systematic review and meta-analysis highlight both the potential and limitations of digital interventions for addressing social isolation and loneliness (SIL). Based on recent (2022-) evidence, while psychological interventions with interactive components, group-based activities, and robotic pets showed promise, self-guided approaches, conversational robots, and social networking and support interventions yielded inconsistent or minimal effects. Notably, digital interventions targeting psychological barriers to social connection—such as cognitive biases and emotional distress—were more effective when they incorporated social elements. Despite these findings, challenges remain. Many studies exhibited moderate to high risk of bias, relied on passive control groups, and lacked long-term follow-up, limiting conclusions about sustained effects. Moving forward, further research is essential to establish a stronger empirical foundation for specific intervention effects, subgroup differences, and long-term outcomes. Key priorities also include replicating the benefits of social and interactive elements, leveraging digital technologies for personalization, using digital tools to support non-digital interventions, systematically comparing digital and non-digital approaches, and, perhaps most critically, examining the impact of increasingly adopted humanlike social chatbots.

## List of abbreviations

NR: Not reported
RoB: Risk of bias
TAU: Treatment-as-usual
WL: Waitlist
NT: No treatment
AT: Alternative treatment
SIL: Social isolation and loneliness
UR: Umbrella review
SR: Systematic review
RCT: Randomized controlled trial
UCLA: University of California, Los Angeles (loneliness scale)
DjG: de Jong Gierveld (loneliness scale)

## Funding

This study was supported by the 10.13039/501100005416Research Council of Norway (grant number 288083).

## Declaration of competing interest

The authors declare that they have no known competing financial interests or personal relationships that could have appeared to influence the work reported in this paper.

## Data Availability

Upon request.

## Appendix A Supplementary data

Supplementary material Image 1
