# Supplementary material for: Digital bridges to social connection: A systematic review and meta-analysis of digital interventions for loneliness and social isolation
Source: Internet Interv. 2025 Jul 8;41:100856. doi: 10.1016/j.invent.2025.100856 (PMC12281013; doi:10.1016/j.invent.2025.100856)

**Appendix**

1. Search strategy (incl. updated search)

2. Prior systematic reviews

3. Excluded studies with reason for exclusion

4. Risk of bias assessment

5. PRISMA form

6. Figures (funnel plot and forest plots)

**APPENDIX 1: SEARCH STRATEGY: RCT’S 2022-2024**

**LONELINESS – digital interventions**

| **Contact person:** | Thomas Hansen |
| --- | --- |
| **Search:** | Ragnhild Agathe Tornes |
| **Peer review:** | Astrid Nøstberg |
| **Duplicate control in Eppi Reviewer:** | Before duplicate control: 28976  After duplicate control: 10475 |
| **Items classified according to model: New Cochrane RCT classifier modelPossibly an RCT:** | 729 |

**Database:** Ovid MEDLINE(R) and Epub Ahead of Print, In-Process, In-Data-Review & Other Non-Indexed Citations, Daily and Versions <1946 to September 24, 2024>

**Date:** 25 September, 2024

**Number of hits:** 5487

| 1 | Loneliness/ or Social Isolation/ or (loneliness or lonely or (social* adj (exclusion? or excluded or isolation or isolated))).tw,kf. | 43309 |
| --- | --- | --- |
| 2 | exp Computers/ or Computers, Handheld/ or Computer User Training/ or Mobile Applications/ or Social Media/ or exp User-Computer Interface/ or Telemedicine/ or Distance Counseling/ or Robotics/ or Wearable Electronic Devices/ or Gamification/ or exp Virtual Reality/ or Internet-Based Intervention/ or Artificial Intelligence/ or ((tele adj (health or med* or psychology or psychiatry or therap* or care or conferenc* or communicat* or consult*)) or telemed* or telepsychology or telepsychiatry or teletherap* or telecare or teleconference* or telecommunicat* or teleconsult* or (social adj (medium? or media? or network* or platform?)) or telehealth or ("e" adj (health or treat* or therap* or counsel* or mail* or support*)) or ehealth or etreat* or etherap* or ecounsel* or email* or support* or m-health or mhealth or ((internet or online or "on line" or cyber or web) adj (based* or treat* or counsel* or therap* or prevention*)) or webcam* or "web cam*" or internetbased or onlinebased or cyberbased or cybercounsel* or video* or skyp* or computer* or electronic* or digital* or technolog* or robot* or telerobotics or audio* or helpline? or ((help or support) adj line?) or ((distance or remote or mobile) adj (counsel* or health)) or text-messag* or textmessag* or messaging or SMS or texting* or short message service? or mobile? or ((smart or cell*) adj phone?) or smartphone? or cellphone? or telephon* or blended* or app or apps or ((handheld or "hand held") adj device?) or iPad* or iPhone* or "i-pad*" or "i-phone*" or tablet* or sensor* or wearable* or palmtop? or "palm top?" or WhatsApp or "Whats App" or Twitter or tweet? or Facebook or facetim* or Instagram or Snapchat or TikTok or "Tik Tok" or WeChat or forum or chat* or (virtual adj (realit* or coach* or character? or human? or assist* or system? or medicine)) or VR or avatar? or ((conversation* or embodied or relational or interactive or virtual) adj agent?) or (serious adj (game? or gaming)) or gamification or ((ICT or "e" or internet or online or "on line" or web) adj intervention?) or "web 2.0*" or "web 3.0*" or wireless or world wide web or AI or "artificial intelligence").tw,kf. | 4775699 |
| 3 | 1 and 2 | 15911 |
| 4 | limit 3 to yr="2022 - Current" | 5487 |

**Database:** Embase <1974 to 2024 September 24>

**Date:** 25 September, 2024

**Number of hits:** 3973

| 1 | loneliness/ or social Isolation/ or (loneliness or lonely or (social* adj (exclusion? or excluded or isolation or isolated))).tw,kf. | 62780 |
| --- | --- | --- |
| 2 | exp computer/ or smartphone/ or smart device/ or human computer interaction/ or exp mobile application/ or social media/ or computer interface/ or telehealth/ or telemedicine/ or telecare/ or exp robotics/ or exp wearable computer/ or gamification/ or virtual reality/ or web-based intervention/ or exp artificial intelligence/ or ((tele adj (health or med* or psychology or psychiatry or therap* or care or conferenc* or communicat* or consult*)) or telemed* or telepsychology or telepsychiatry or teletherap* or telecare or teleconference* or telecommunicat* or teleconsult* or (social adj (medium? or media? or network* or platform?)) or telehealth or ("e" adj (health or treat* or therap* or counsel* or mail* or support*)) or ehealth or etreat* or etherap* or ecounsel* or email* or support* or m-health or mhealth or ((internet or online or "on line" or cyber or web) adj (based* or treat* or counsel* or therap* or prevention*)) or webcam* or "web cam*" or internetbased or onlinebased or cyberbased or cybercounsel* or video* or skyp* or computer* or electronic* or digital* or technolog* or robot* or telerobotics or audio* or helpline? or ((help or support) adj line?) or ((distance or remote or mobile) adj (counsel* or health)) or text-messag* or textmessag* or messaging or SMS or texting* or short message service? or mobile? or ((smart or cell*) adj phone?) or smartphone? or cellphone? or telephon* or blended* or app or apps or ((handheld or "hand held") adj device?) or iPad* or iPhone* or "i-pad*" or "i-phone*" or tablet* or sensor* or wearable* or palmtop? or "palm top?" or WhatsApp or "Whats App" or Twitter or tweet? or Facebook or facetim* or Instagram or Snapchat or TikTok or "Tik Tok" or WeChat or forum or chat* or (virtual adj (realit* or coach* or character? or human? or assist* or system? or medicine)) or VR or avatar? or ((conversation* or embodied or relational or interactive or virtual) adj agent?) or (serious adj (game? or gaming)) or gamification or ((ICT or "e" or internet or online or "on line" or web) adj intervention?) or "web 2.0*" or "web 3.0*" or wireless or world wide web or AI or "artificial intelligence").tw,kf. | 6146100 |
| 3 | 1 and 2 | 22912 |
| 4 | limit 3 to yr="2022 -Current" | 7192 |
| 5 | limit 4 to (embase or "preprints (unpublished, non-peer reviewed)") | 3973 |

**Database:** APA PsycInfo <1806 to September 2024 Week 3>

**Date:** 25 September, 2024

**Number of hits:** 3324

| 1 | Loneliness/ or Social Isolation/ or (loneliness or lonely or (social* adj (exclusion? or excluded or isolation or isolated))).tw. | 36552 |
| --- | --- | --- |
| 2 | exp Computers/ or Human Computer Interaction/ or Mobile Applications/ or exp Social Media/ or exp Human Computer Interaction/ or exp Telemedicine/ or Digital Interventions/ or Electronic Health Services/ or Mobile Health/ or Wearable Devices/ or Games/ or Computer Games/ or Computer Applications/ or Digital Gaming/ or Smartphones/ or Virtual Reality/ or Virtual Reality Exposure Therapy/ or exp Artificial Intelligence/ or ((tele adj (health or med* or psychology or psychiatry or therap* or care or conferenc* or communicat* or consult*)) or telemed* or telepsychology or telepsychiatry or teletherap* or telecare or teleconference* or telecommunicat* or teleconsult* or (social adj (medium? or media? or network* or platform?)) or telehealth or ("e" adj (health or treat* or therap* or counsel* or mail* or support*)) or ehealth or etreat* or etherap* or ecounsel* or email* or support* or m-health or mhealth or ((internet or online or "on line" or cyber or web) adj (based* or treat* or counsel* or therap* or prevention*)) or webcam* or "web cam*" or internetbased or onlinebased or cyberbased or cybercounsel* or video* or skyp* or computer* or electronic* or digital* or technolog* or robot* or telerobotics or audio* or helpline? or ((help or support) adj line?) or ((distance or remote or mobile) adj (counsel* or health)) or text-messag* or textmessag* or messaging or SMS or texting* or short message service? or mobile? or ((smart or cell*) adj phone?) or smartphone? or cellphone? or telephon* or blended* or app or apps or ((handheld or "hand held") adj device?) or iPad* or iPhone* or "i-pad*" or "i-phone*" or tablet* or sensor* or wearable* or palmtop? or "palm top?" or WhatsApp or "Whats App" or Twitter or tweet? or Facebook or facetim* or Instagram or Snapchat or TikTok or "Tik Tok" or WeChat or forum or chat* or (virtual adj (realit* or coach* or character? or human? or assist* or system? or medicine)) or VR or avatar? or ((conversation* or embodied or relational or interactive or virtual) adj agent?) or (serious adj (game? or gaming)) or gamification or ((ICT or "e" or internet or online or "on line" or web) adj intervention?) or "web 2.0*" or "web 3.0*" or wireless or world wide web or AI or "artificial intelligence").tw. | 1442711 |
| 3 | 1 and 2 | 14121 |
| 4 | limit 3 to yr="2022 -Current" | 3324 |

**Database: Sociological Abstracts via ProQuest**

**Date:** 25 September, 2024

**Number of hits:** 4,211

| S1 | Searched for: SU.EXACT("Loneliness" or "Social Isolation") | 154 |
| --- | --- | --- |
| S2 | [STRICT] AB,TI(loneliness or lonely or (social* P/0 (exclusion or exclusions or excluded or isolation or isolated))) | 16,189 |
| S3 | [S1] OR [S2] | 16,236 |
| S4 | SU.EXACT("Computers" or "Mobile phones" or "Human-computer interaction" or "Social media" or "Virtual reality" or "Artificial intelligence") | 16,147 |
| S5 | [STRICT] AB,TI((tele P/0 (health OR med* OR psychology OR psychiatry OR therap* OR care OR conferenc* OR communicat* OR consult*)) OR telehealth OR telemed* OR telepsychology OR telepsychiatry OR teletherap* OR telecare OR teleconference* OR telecommunicat* OR teleconsult* OR (social P/0 (medium OR mediums OR media OR medias OR network* OR platform OR platforms)) OR ("e" P/0 (health OR treat* OR therap* OR counsel* OR mail* OR support*)) OR ehealth OR etreat* or etherap* OR ecounsel* OR email* OR support* OR "m-health" OR mhealth OR ((internet OR online OR "on line" OR cyber OR web) P/0 (based* OR treat* OR counsel* OR therap* OR prevention*)) OR webcam* OR "web cam*" OR internetbased OR onlinebased OR cyberbased OR cybercounsel* OR video* OR skyp* OR computer* OR electronic* OR digital* OR technolog* OR robot* OR telerobotics OR audio* OR helpline* OR ((help OR support) P/0 line*) OR ((distance OR remote OR mobile) P/0 (counsel* OR health)) OR "text-messag*" OR textmessag* OR messaging OR SMS OR texting* OR "short message service*" OR mobile OR mobiles OR ((smart OR cell*) P/0 phone*) OR smartphone* OR cellphone* OR telephon* OR blended* OR app OR apps OR ((handheld OR "hand held") P/0 device*) OR iPad* or iPhone* or "i-pad*" or "i-phone*" OR tablet* OR sensor* OR wearable* OR palmtop* OR "palm top*" OR WhatsApp or "Whats App" OR Twitter OR tweet OR tweets OR Facebook OR facetim* OR Instagram OR Snapchat or TikTok or "Tik Tok" or WeChat OR forum OR chat* OR (virtual P/0 (realit* OR coach* OR character OR characters OR human OR humans OR assist* OR system OR systems OR medicine)) OR VR OR avatar* OR ((conversation* OR embodied OR relational OR interactive OR virtual) P/0 (agent OR agents)) OR (serious P/0 (game OR games OR gaming)) OR gamification OR ((ICT OR "e" OR internet OR online OR "on line" OR web) P/0 intervention*) OR "web 2.0*" OR "web 3.0*" OR wireless OR "world wide web" OR AI OR "artificial intelligence") | 256,194 |
| S6 | [S3] OR [S4] | 488,748 |
| S7 | [S3] AND [S6] | 16,147 |
| S8 | [S3] AND [S6]  Limits applied: Publication date 2022-01-01 to 2024-09-24 | 4,211 |

**Database: Web of Science Core Collection**

**Science Citation Index Expanded**

**(SCI-EXPANDED)--1987-present**

**Social Sciences Citation Index**

**(SSCI)--1987-present**

**Arts & Humanities Citation Index**

**(AHCI)--1987-present**

**Emerging Sources Citation Index**

**(ESCI)—2019-present**

**Date:** 25 September, 2024

**Number of hits:** 8491

| 1 | TS=(loneliness or lonely or (social* NEAR/0 (exclusion$ or excluded or isolation or isolated))) | exact search | 54877 |
| --- | --- | --- | --- |
| 2 | TS=((tele NEAR/0 (health or med* or psychology or psychiatry or therap* or care or conferenc* or communicat* or consult*)) or telehealth or telemed* or telepsychology or telepsychiatry or teletherap* or telecare or teleconference* or telecommunicat* or teleconsult* or (social NEAR/0 (medium$ or media$ or network* or platform$)) or ("e" NEAR/0 (health or treat* or therap* or counsel* or mail* or support*)) or ehealth or etreat* or etherap* or ecounsel* or email* or support* or "m-health" or mhealth or ((internet or online or "on line" or cyber or web) NEAR/0 (based* or treat* or counsel* or therap* or prevention*)) or webcam* or "web cam*" or internetbased or onlinebased or cyberbased or cybercounsel* or video* or skyp* or computer* or electronic* or digital* or technolog* or robot* or telerobotics or audio* or helpline$ or ((help or support) NEAR/0 line$) or ((distance or remote or mobile) NEAR/0 (counsel* or health)) or "text-messag*" or textmessag* or messaging or SMS or texting* or "short message service$" or mobile$ or ((smart or cell*) NEAR/0 phone$) or smartphone$ or cellphone$ or telephon* or blended* or app or apps or ((handheld or "hand held") NEAR/0 device$) or iPad* or iPhone* or "i-pad*" or "i-phone*" or tablet* or sensor* or wearable* or palmtop$ or "palm top$" or WhatsApp or "Whats App" or Twitter or tweet$ or Facebook or facetim* or Instagram or Snapchat or TikTok or "Tik Tok" or WeChat or forum or chat* or (virtual NEAR/0 (realit* or coach* or character$ or human$ or assist* or system$ or medicine)) or VR or avatar$ or ((conversation* or embodied or relational or interactive or virtual) NEAR/0 agent$) or (serious NEAR/0 (game$ or gaming)) or gamification or ((ICT or "e" or internet or online or "on line" or web) NEAR/0 intervention$) or "web 2.0*" or "web 3.0*" or wireless or "world wide web" or AI or "artificial intelligence") | exact search | 9577077 |
| 3 | #1 AND #2 | exact search | 23667 |
| 4 | #3  Timespan: 2022-01-01 to 2024-09-25 | exact search | 8491 |

**Database: Cinahl via Ebsco**

**Date:** 25 September, 2024

**Number of hits:** 2799

| S1 | (MH "Loneliness") or (MH "Social Isolation") | (17,940) |
| --- | --- | --- |
| S2 | TI(loneliness or lonely or (social* W0 (exclusion# or excluded or isolation or isolated))) OR AB (loneliness or lonely or (social* W0 (exclusion# or excluded or isolation or isolated))) | (16,168) |
| S3 | S1 OR S2 | (25,752) |
| S4 | (MH "Computers, Portable") or (MH "Computers and Computerization") or (MH "Computers, Hand-Held") or (MH "User-Computer Interface") or (MH "Mobile Applications") or (MH "Social Media") or (MH "Twitter") or (MH "Telemedicine") or (MH "Telerehabilitation") or (MH "Telehealth") or (MH "Telepsychiatry") or (MH "Robotics") or (MH "Virtual Reality") or (MH "Virtual Reality Exposure Therapy") or (MH "Smartphone") or (MH "Text Messaging") or (MH "Cellular Phone") or (MH "Artificial Intelligence") | (127,724) |
| S5 | TI((tele W0 (health or med* or psychology or psychiatry or therap* or care or conferenc* or communicat* or consult*)) OR telehealth OR telemed* or telepsychology OR telepsychiatry OR teletherap* OR telecare OR teleconference* OR telecommunicat* OR teleconsult* or (social W0 (medium# or media# or network* or platform#)) OR ("e" W0 (health or treat* or therap* or counsel* or mail* or support*)) or ehealth OR etreat* or etherap* or ecounsel* OR email* OR support* OR "m-health" OR mhealth OR ((internet or online or "on line" or cyber or web) W0 (based* or treat* or counsel* or therap* or prevention*)) or webcam* or "web cam*" or internetbased or onlinebased or cyberbased OR cybercounsel* or video* or skyp* or computer* OR electronic* OR digital* OR technolog* OR robot* or telerobotics or audio* or helpline# or ((help or support) W0 line#) or ((distance or remote or mobile) W0 (counsel* or health)) OR "text-messag*" OR textmessag* or messaging OR SMS OR texting* OR "short message service#" OR mobile# OR ((smart or cell*) W0 phone#) OR smartphone# or cellphone# OR telephon* or blended* OR app OR apps OR ((handheld or "hand held") W0 device#) OR iPad* or iPhone* or "i-pad*" or "i-phone*" OR tablet* OR sensor* OR wearable* OR palmtop# OR "palm top#" OR WhatsApp or "Whats App" OR Twitter or tweet# OR Facebook or facetim* OR Instagram OR Snapchat or TikTok or "Tik Tok" or WeChat OR forum OR chat* OR (virtual W0 (realit* or coach* or character# or human# or assist* or system# or medicine)) OR VR OR avatar# OR ((conversation* or embodied or relational or interactive or virtual) W0 agent#) OR (serious W0 (game# OR gaming)) OR gamification OR ((ICT or "e" or internet or online or "on line" or web) W0 intervention#) or "web 2.0*" or "web 3.0*" or wireless or "world wide web" or AI or "artificial intelligence") OR AB((tele W0 (health or med* or psychology or psychiatry or therap* or care or conferenc* or communicat* or consult*)) OR telehealth OR telemed* or telepsychology OR telepsychiatry OR teletherap* OR telecare OR teleconference* OR telecommunicat* OR teleconsult* or (social W0 (medium# or media# or network* or platform#)) OR ("e" W0 (health or treat* or therap* or counsel* or mail* or support*)) or ehealth OR etreat* or etherap* or ecounsel* OR email* OR support* OR "m-health" OR mhealth OR ((internet or online or "on line" or cyber or web) W0 (based* or treat* or counsel* or therap* or prevention*)) or webcam* or "web cam*" or internetbased or onlinebased or cyberbased OR cybercounsel* or video* or skyp* or computer* OR electronic* OR digital* OR technolog* OR robot* or telerobotics or audio* or helpline# or ((help or support) W0 line#) or ((distance or remote or mobile) W0 (counsel* or health)) OR "text-messag*" OR textmessag* or messaging OR SMS OR texting* OR "short message service#" OR mobile# OR ((smart or cell*) W0 phone#) OR smartphone# or cellphone# OR telephon* or blended* OR app OR apps OR ((handheld or "hand held") W0 device#) OR iPad* or iPhone* or "i-pad*" or "i-phone*" OR tablet* OR sensor* OR wearable* OR palmtop# OR "palm top#" OR WhatsApp or "Whats App" OR Twitter or tweet# OR Facebook or facetim* OR Instagram OR Snapchat or TikTok or "Tik Tok" or WeChat OR forum OR chat* OR (virtual W0 (realit* or coach* or character# or human# or assist* or system# or medicine)) OR VR OR avatar# OR ((conversation* or embodied or relational or interactive or virtual) W0 agent#) OR (serious W0 (game# OR gaming)) OR gamification OR ((ICT or "e" or internet or online or "on line" or web) W0 intervention#) or "web 2.0*" or "web 3.0*" or wireless or "world wide web" or AI or "artificial intelligence") | (1,159,408) |
| S6 | S4 OR S5 | (1,198,242) |
| S7 | S3 AND S6 | (9,861) |
| S8 | S3 AND S6  Limiters - Publication Date: 20220101-20241231 | (2,986) |
| S9 | S8  Exclude MEDLINE records | (2,799) |

**Database:** Cochrane Central Register of Controlled Trials

Issue 8 of 12, August 2024

**Date:** 25 September, 2024

**Number of hits:** 691

| #1 | [mh ^Loneliness] or [mh ^"Social Isolation"] | 503 |
| --- | --- | --- |
| #2 | (loneliness or lonely or (social* NEXT (exclusion? or excluded or isolation or isolated))):ti,ab | 2579 |
| #3 | #1 or #2 | 2746 |
| #4 | [mh Computers] or [mh "Computers, Handheld"] or [mh ^"Computer User Training"] or [mh ^"Mobile Applications"] or [mh ^"Social Media"] or [mh "User-Computer Interface"] or [mh ^Telemedicine] or [mh ^"Distance Counseling"] or [mh ^Robotics] or [mh ^"Wearable Electronic Devices"] or [mh ^Gamification] or [mh "Virtual Reality"] or [mh ^"Internet-Based Intervention"] or [mh "Artificial Intelligence"] | 15269 |
| #5 | ((tele NEXT (health or med* or psychology or psychiatry or therap* or care or conferenc* or communicat* or consult*)) OR telehealth OR telemed* or telepsychology OR telepsychiatry OR teletherap* OR telecare OR teleconference* OR telecommunicat* OR teleconsult* or (social NEXT (medium? or media? or network* or platform?)) OR ("e" NEXT (health or treat* or therap* or counsel* or mail* or support*)) or ehealth OR etreat* or etherap* or ecounsel* OR email* OR support* OR "m-health" OR mhealth OR ((internet or online or "on line" or cyber or web) NEXT (based* or treat* or counsel* or therap* or prevention*)) or webcam* or (web NEXT cam*) or internetbased or onlinebased or cyberbased OR cybercounsel* or video* or skyp* or computer* OR electronic* OR digital* OR technolog* OR robot* or telerobotics or audio* or helpline? or ((help or support) NEXT line?) or ((distance or remote or mobile) NEXT (counsel* or health)) OR (text NEXT messag*) OR textmessag* or messaging OR SMS OR texting* OR ("short message" NEXT service?) OR mobile? OR ((smart or cell*) NEXT phone?) OR smartphone? or cellphone? OR telephon* or blended* OR app OR apps OR ((handheld or "hand held") NEXT device?) OR iPad* or iPhone* or ("i" NEXT (pad* or phone*)) OR tablet* OR sensor* OR wearable* OR palmtop? or (palm NEXT top?) OR WhatsApp or "Whats App" OR Twitter or tweet? OR Facebook or facetim* OR Instagram OR Snapchat OR TikTok OR "Tik Tok" OR WeChat OR forum OR chat* OR (virtual NEXT (realit* or coach* or character? or human? or assist* or system? or medicine)) OR VR OR avatar? OR ((conversation* or embodied or relational or interactive or virtual) NEXT agent?) OR (serious NEXT (game? OR gaming)) OR gamification OR ((ICT or "e" or internet or online or "on line" or web) NEXT intervention?) or (web NEXT ("2.0" or "3.0")) or wireless or "world wide web" or AI or "artificial intelligence"):ti,ab | 441908 |
| #6 | #4 or #5 | 443840 |
| #7 | #3 and #6 | 1682 |
| #8 | #7 with Cochrane Library publication date Between Jan 2022 and Sep 2024, in Trials | 679 |
| #9 | #7 with Publication Year from 2022 to 2024, in Trials | 594 |
| #10 | #8 or #9 | 691 |

**APPENDIX 1B: SEARCH STRATEGY: GREY LITERATURE 2022-2024**

Loneliness and social isolation

Effect of digital interventions against loneliness and social isolation

**Contact person:** Thomas Hansen

**Search:** Ragnhild Agathe Tornes

**Date:** 7 and 9 October 2024

**Number of matches before duplicate control:** 467

**Number of matches after duplicate control:** 391 (in addition, look at links for hits that could not be exported)

| DATABASE | DATO | SØK | Treff |
| --- | --- | --- | --- |
| Swemed+  <https://svemedplus.kib.ki.se/> | -- | \| Hasn't been updated since 2019, so nothing to pick up here. \| Antall träffar \| \| --- \| --- \| | --- |
| ClinicalTrials.gov  [www.clinicaltrials.gov](http://www.clinicaltrials.gov) | 9 October 2024 | Loneliness  Hitlist: <https://www.clinicaltrials.gov/search?cond=Loneliness>  Social isolation  Hitlist: <https://www.clinicaltrials.gov/search?cond=Social%20Isolation> | 241  202 |
| Open Grey System for Information on Grey Literature in Europe  [https://easy.dans.knaw.nl/ui/advancedsearch](https://easy.dans.knaw.nl/ui/advancedsearch;jsessionid=526DDB7F427C278D2DA1D35A6666CB7F) | 9 October 2024 | Advanced search: Any field: (loneliness or lonely or "social exclusion" or "social exclusions" or "socially excluded" or "social isolation" or "socially isolated")  164 hits, but only 1 in the time space 2022-2024  Link to the single hit: <https://lifesciences.datastations.nl/dataset.xhtml?persistentId=doi:10.17026/dans-zqn-zgex> | 1 |
| SBU – Statens beredning för medicinsk och social utvärdering  <https://www.sbu.se/sv/> | 7 October 2024 | The results cannot be exported. Look through the hit lists (Choose "Sortera efter: Datum" to get the latest hits first):  ensamhet: <https://www.sbu.se/sv/sok/?q=ensamhet&p=1&s=1&ps=10>  social isolering: <https://www.sbu.se/sv/sok/?q=social%20isolering&p=1&s=0&ps=10> | sjå lenker |
| WHO Library Catalog  <https://kohahq.searo.who.int/cgi-bin/koha/opac-search.pl> | 7 October 2024  TECHNICAL ERROR, COULD NOT PERFORM SEARCH | (Advanced search:  Keyword: loneliness  Publication date range: 2022-2024) |  |
| WHO IRIS  <http://apps.who.int/iris/> | 9 October 2024 | Browsing by Subject "Loneliness"  Browsing by Subject "Social isolation" | 4  (0 from 2022-2024)  9  (0 from 2022-2024) |
| [Mednar](https://mednar.com/mednar/desktop/en/search.html)  <https://mednar.com/mednar/desktop/en/search.html> | 9 October 2024 | Advanced search: Full Record: loneliness / From: 2022 / To: 2024  Limit to Medical | 24 |
| [Socialstyrelsen](https://socialstyrelsen.dk/udgivelser) | 10/10-24 | Search: ensamhet  172 results with this limitation:  Innholdstyp: Publikationer  Sort on: Datum" and browse through the ones from 2022-2024:  <https://www.socialstyrelsen.se/sok/?q=ensamhet> | sjå lenke |
| [Statens Institut for Folkesundhed](https://www.sdu.dk/da/sif/rapporter) | 7 October 2024 | Search: ensomhed  99 hits  Check the 99 hits to find publications from 2022 until current:  <https://www.sdu.dk/da/sif/search?q=ensomhed&l=0&ps=&c=&sc=&s=1&pst=> | sjå lenke |

**SEARCH STRATEGY: REVIEWS 2022-2024**

**LONELINESS – digital interventions**

**– SYSTEMATIC REVIEWS**

| **Contact person:** | Thomas Hansen |
| --- | --- |
| **Search:** | Ragnhild Agathe Tornes |
| **Duplicate check in EndNote:** | Before Duplicate check: 659  After Duplicate check: 449 |

Ovid MEDLINE(R) and Epub Ahead of Print, In-Process, In-Data-Review & Other Non-Indexed Citations, Daily and Versions <1946 to September 17, 2024>

| 1 | Loneliness/ or Social Isolation/ | 22593 |
| --- | --- | --- |
| 2 | (loneliness or lonely or (social* adj (exclusion? or excluded or isolation or isolated))).tw,kf. | 31650 |
| 3 | 1 or 2 | 43221 |
| 4 | exp Computers/ or exp Computers, Handheld/ or Computer User Training/ or Mobile Applications/ or Social Media/ or exp User-Computer Interface/ or Telemedicine/ or Distance Counseling/ or Robotics/ or Wearable Electronic Devices/ or Gamification/ or exp Virtual Reality/ or Internet-Based Intervention/ or exp Artificial Intelligence/ | 405623 |
| 5 | ((tele adj (health or med* or psychology or psychiatry or therap* or care or conferenc* or communicat* or consult*)) or telehealth or telemed* or telepsychology or telepsychiatry or teletherap* or telecare or teleconference* or telecommunicat* or teleconsult* or (social adj (medium? or media? or network* or platform?)) or telehealth or ("e" adj (health or treat* or therap* or counsel* or mail* or support*)) or ehealth or etreat* etherap* or ecounsel* or email* or m-health or mhealth or ((internet or online or cyber or web) adj (based* or treat* or councel* or therap* or prevention*)) or webcam* or "web cam*" or internetbased or onlinebased or cyberbased or cybercounsel* or video* or skyp* or computer* or electronic* or digital* or technolog* or robot* or telerobotics or audio* or helpline? or ((help or support) adj line?) or ((distance or remote or mobile) adj (counsel* or health)) or text-messag* or textmessag* or messaging or SMS or texting* or short message service? or mobile? or ((smart or cell*) adj phone?) or smartphone? or cellphone? or telephon* or blended* or app or apps or ((handheld or "hand held") adj device?) or iPad* or iPhone* or tablet* or sensor* or wearable* or palmtop? or WhatsApp or Twitter or tweet? or Facebook or facetim* or Instagram or Snapchat or TikTok or WeChat or WhatsApp or forum or chat* or (virtual adj (realit* or coach* or character? or human? or assist* or system? or medicine)) or VR or avatar? or ((conversation* or embodied or relational or interactive or virtual) adj agent?) or (serious adj (game? or gaming)) or gamification or ((ICT or "e" or internet or online or web) adj intervention?) or "web 2.0*" or "web 3.0*" or wireless or world wide web or AI or "artificial intelligence").tw,kf. | 2887994 |
| 6 | 4 or 5 | 3056594 |
| 7 | 3 and 6 | 8042 |
| 8 | limit 7 to yr="2022 -Current" | 3078 |
| 9 | limit 8 to "reviews (maximizes specificity)" | 219 |
| 10 | Meta-Analysis/ or Network Meta-Analysis/ or ((systematic* adj2 review*) or metaanal* or "meta anal*" or (review and ((structured or database* or systematic*) adj2 search*)) or "integrative review*" or (evidence adj2 review*)).tw,kf,bt. | 588230 |
| 11 | 9 or (8 and 10) | 285 |

Embase <1974 to 2024 September 17>

| 1 | loneliness/ or social Isolation/ | 48580 |
| --- | --- | --- |
| 2 | (loneliness or lonely or (social* adj (exclusion? or excluded or isolation or isolated))).tw,kf. | 39025 |
| 3 | 1 or 2 | 62662 |
| 4 | exp computer/ or smartphone/ or smart device/ or human computer interaction/ or exp mobile application/ or social media/ or computer interface/ or telehealth/ or telemedicine/ or telecare/ or exp robotics/ or exp wearable computer/ or gamification/ or virtual reality/ or web-based intervention/ or exp artificial intelligence/ | 548556 |
| 5 | ((tele adj (health or med* or psychology or psychiatry or therap* or care or conferenc* or communicat* or consult*)) or telehealth or telemed* or telepsychology or telepsychiatry or teletherap* or telecare or teleconference* or telecommunicat* or teleconsult* or (social adj (medium? or media? or network* or platform?)) or telehealth or ("e" adj (health or treat* or therap* or counsel* or mail* or support*)) or ehealth or etreat* etherap* or ecounsel* or email* or m-health or mhealth or ((internet or online or cyber or web) adj (based* or treat* or councel* or therap* or prevention*)) or webcam* or "web cam*" or internetbased or onlinebased or cyberbased or cybercounsel* or video* or skyp* or computer* or electronic* or digital* or technolog* or robot* or telerobotics or audio* or helpline? or ((help or support) adj line?) or ((distance or remote or mobile) adj (counsel* or health)) or text-messag* or textmessag* or messaging or SMS or texting* or short message service? or mobile? or ((smart or cell*) adj phone?) or smartphone? or cellphone? or telephon* or blended* or app or apps or ((handheld or "hand held") adj device?) or iPad* or iPhone* or tablet* or sensor* or wearable* or palmtop? or WhatsApp or Twitter or tweet? or Facebook or facetim* or Instagram or Snapchat or TikTok or WeChat or WhatsApp or forum or chat* or (virtual adj (realit* or coach* or character? or human? or assist* or system? or medicine)) or VR or avatar? or ((conversation* or embodied or relational or interactive or virtual) adj agent?) or (serious adj (game? or gaming)) or gamification or ((ICT or "e" or internet or online or web) adj intervention?) or "web 2.0*" or "web 3.0*" or wireless or world wide web or AI or "artificial intelligence").tw,kf. | 3669199 |
| 6 | 4 or 5 | 3820320 |
| 7 | 3 and 6 | 11783 |
| 8 | limit 7 to yr="2022 -Current" | 4129 |
| 9 | limit 8 to "reviews (maximizes specificity)" | 209 |
| 10 | exp Meta-Analysis/ or "systematic review"/ or ((systematic* adj2 review*) or metaanal* or "meta anal*" or (review and ((structured or database* or systematic*) adj2 search*)) or "integrative review*" or (evidence adj2 review*)).tw,kf,bt. | 843217 |
| 11 | 9 or (8 and 10) | 346 |
| 12 | limit 11 to (embase or "preprints (unpublished, non-peer reviewed)") | 218 |

APA PsycInfo <1806 to September 2024 Week 2>

| 1 | Loneliness/ or Social Isolation/ | 16802 |
| --- | --- | --- |
| 2 | (loneliness or lonely or (social* adj (exclusion? or excluded or isolation or isolated))).tw. | 32230 |
| 3 | 1 or 2 | 36508 |
| 4 | exp Computers/ or Human Computer Interaction/ or Mobile Applications/ or exp Social Media/ or exp Human Computer Interaction/ or exp Telemedicine/ or Digital Interventions/ or Electronic Health Services/ or Mobile Health/ or Wearable Devices/ or Games/ or Computer Games/ or Computer Applications/ or Digital Gaming/ or Smartphones/ or Virtual Reality/ or Virtual Reality Exposure Therapy/ or exp Artificial Intelligence/ | 207954 |
| 5 | ((tele adj (health or med* or psychology or psychiatry or therap* or care or conferenc* or communicat* or consult*)) or telehealth or telemed* or telepsychology or telepsychiatry or teletherap* or telecare or teleconference* or telecommunicat* or teleconsult* or (social adj (medium? or media? or network* or platform?)) or telehealth or ("e" adj (health or treat* or therap* or counsel* or mail* or support*)) or ehealth or etreat* etherap* or ecounsel* or email* or m-health or mhealth or ((internet or online or cyber or web) adj (based* or treat* or councel* or therap* or prevention*)) or webcam* or "web cam*" or internetbased or onlinebased or cyberbased or cybercounsel* or video* or skyp* or computer* or electronic* or digital* or technolog* or robot* or telerobotics or audio* or helpline? or ((help or support) adj line?) or ((distance or remote or mobile) adj (counsel* or health)) or text-messag* or textmessag* or messaging or SMS or texting* or short message service? or mobile? or ((smart or cell*) adj phone?) or smartphone? or cellphone? or telephon* or blended* or app or apps or ((handheld or "hand held") adj device?) or iPad* or iPhone* or tablet* or sensor* or wearable* or palmtop? or WhatsApp or Twitter or tweet? or Facebook or facetim* or Instagram or Snapchat or TikTok or WeChat or WhatsApp or forum or chat* or (virtual adj (realit* or coach* or character? or human? or assist* or system? or medicine)) or VR or avatar? or ((conversation* or embodied or relational or interactive or virtual) adj agent?) or (serious adj (game? or gaming)) or gamification or ((ICT or "e" or internet or online or web) adj intervention?) or "web 2.0*" or "web 3.0*" or wireless or world wide web or AI or "artificial intelligence").tw. | 672350 |
| 6 | 4 or 5 | 736362 |
| 7 | 3 and 6 | 6493 |
| 8 | limit 7 to yr="2022 -Current" | 1730 |
| 9 | limit 8 to "reviews (maximizes specificity)" | 129 |
| 10 | (meta analysis or "systematic review").md. or meta analysis/ or ((systematic* adj2 review*) or metaanal* or "meta anal*" or (review and ((structured or database* or systematic*) adj2 search*)) or "integrative review*" or (evidence adj2 review*)).tw. | 124753 |
| 11 | 9 or (8 and 10) | 156 |

**LONELINESS – digital interventions – updated search**

| **Contact person:** | Thomas Hansen |
| --- | --- |
| **Search:** | Ragnhild Agathe Tornes |
| **Duplicate control in Eppi Reviewer:** | Before duplicate control: 3596  After duplicate control: 2323 |
| **Items classified according to model: New Cochrane RCT classifier model: Possibly an RCT:** | 138 |

###

**Database:** Ovid MEDLINE(R) and Epub Ahead of Print, In-Process, In-Data-Review & Other Non-Indexed Citations, Daily and Versions <1946 to January 31, 2025>

**Date:** 03 February 2025

**Number of hits:** 1102

| 1 | Loneliness/ or Social Isolation/ or (loneliness or lonely or (social* adj (exclusion? or excluded or isolation or isolated))).tw,kf. | 44784 |
| --- | --- | --- |
| 2 | exp Computers/ or Computers, Handheld/ or Computer User Training/ or Mobile Applications/ or Social Media/ or exp User-Computer Interface/ or Telemedicine/ or Distance Counseling/ or Robotics/ or Wearable Electronic Devices/ or Gamification/ or exp Virtual Reality/ or Internet-Based Intervention/ or Artificial Intelligence/ or ((tele adj (health or med* or psychology or psychiatry or therap* or care or conferenc* or communicat* or consult*)) or telemed* or telepsychology or telepsychiatry or teletherap* or telecare or teleconference* or telecommunicat* or teleconsult* or (social adj (medium? or media? or network* or platform?)) or telehealth or ("e" adj (health or treat* or therap* or counsel* or mail* or support*)) or ehealth or etreat* or etherap* or ecounsel* or email* or support* or m-health or mhealth or ((internet or online or "on line" or cyber or web) adj (based* or treat* or counsel* or therap* or prevention*)) or webcam* or "web cam*" or internetbased or onlinebased or cyberbased or cybercounsel* or video* or skyp* or computer* or electronic* or digital* or technolog* or robot* or telerobotics or audio* or helpline? or ((help or support) adj line?) or ((distance or remote or mobile) adj (counsel* or health)) or text-messag* or textmessag* or messaging or SMS or texting* or short message service? or mobile? or ((smart or cell*) adj phone?) or smartphone? or cellphone? or telephon* or blended* or app or apps or ((handheld or "hand held") adj device?) or iPad* or iPhone* or "i-pad*" or "i-phone*" or tablet* or sensor* or wearable* or palmtop? or "palm top?" or WhatsApp or "Whats App" or Twitter or tweet? or Facebook or facetim* or Instagram or Snapchat or TikTok or "Tik Tok" or WeChat or forum or chat* or (virtual adj (realit* or coach* or character? or human? or assist* or system? or medicine)) or VR or avatar? or ((conversation* or embodied or relational or interactive or virtual) adj agent?) or (serious adj (game? or gaming)) or gamification or ((ICT or "e" or internet or online or "on line" or web) adj intervention?) or "web 2.0*" or "web 3.0*" or wireless or world wide web or AI or "artificial intelligence").tw,kf. | 4911934 |
| 3 | 1 and 2 | 16705 |
| 4 | 2025*.ed,ep,yr,dp,dt. | 229400 |
| 5 | (202409* or 202410* or 202411* or 202412*).ep,ed,dt. | 671376 |
| 6 | 3 and (4 or 5) | 1102 |

**Database:** Embase <1974 to 2025 January 31>

**Date:** 03 February 2025

**Number of hits:** 513

| 1 | loneliness/ or social Isolation/ or (loneliness or lonely or (social* adj (exclusion? or excluded or isolation or isolated))).tw,kf. | 63988 |
| --- | --- | --- |
| 2 | exp computer/ or smartphone/ or smart device/ or human computer interaction/ or exp mobile application/ or social media/ or computer interface/ or telehealth/ or telemedicine/ or telecare/ or exp robotics/ or exp wearable computer/ or gamification/ or virtual reality/ or web-based intervention/ or exp artificial intelligence/ or ((tele adj (health or med* or psychology or psychiatry or therap* or care or conferenc* or communicat* or consult*)) or telemed* or telepsychology or telepsychiatry or teletherap* or telecare or teleconference* or telecommunicat* or teleconsult* or (social adj (medium? or media? or network* or platform?)) or telehealth or ("e" adj (health or treat* or therap* or counsel* or mail* or support*)) or ehealth or etreat* or etherap* or ecounsel* or email* or support* or m-health or mhealth or ((internet or online or "on line" or cyber or web) adj (based* or treat* or counsel* or therap* or prevention*)) or webcam* or "web cam*" or internetbased or onlinebased or cyberbased or cybercounsel* or video* or skyp* or computer* or electronic* or digital* or technolog* or robot* or telerobotics or audio* or helpline? or ((help or support) adj line?) or ((distance or remote or mobile) adj (counsel* or health)) or text-messag* or textmessag* or messaging or SMS or texting* or short message service? or mobile? or ((smart or cell*) adj phone?) or smartphone? or cellphone? or telephon* or blended* or app or apps or ((handheld or "hand held") adj device?) or iPad* or iPhone* or "i-pad*" or "i-phone*" or tablet* or sensor* or wearable* or palmtop? or "palm top?" or WhatsApp or "Whats App" or Twitter or tweet? or Facebook or facetim* or Instagram or Snapchat or TikTok or "Tik Tok" or WeChat or forum or chat* or (virtual adj (realit* or coach* or character? or human? or assist* or system? or medicine)) or VR or avatar? or ((conversation* or embodied or relational or interactive or virtual) adj agent?) or (serious adj (game? or gaming)) or gamification or ((ICT or "e" or internet or online or "on line" or web) adj intervention?) or "web 2.0*" or "web 3.0*" or wireless or world wide web or AI or "artificial intelligence").tw,kf. | 6237893 |
| 3 | 1 and 2 | 23574 |
| 4 | limit 3 to (embase or "preprints (unpublished, non-peer reviewed)") | 12478 |
| 5 | 2025*.yr,dd,dp,dc. | 232496 |
| 6 | (202409* or 202410* or 202411* or 202412*).dd,dc. | 671613 |
| 7 | 4 and (5 or 6) | 513 |

**Database:** APA PsycInfo <1806 to January 2025 Week 4>

**Date:** 04 February 2025

**Number of hits:** 593

| 1 | Loneliness/ or Social Isolation/ or (loneliness or lonely or (social* adj (exclusion? or excluded or isolation or isolated))).tw. | 37559 |
| --- | --- | --- |
| 2 | exp Computers/ or Human Computer Interaction/ or Mobile Applications/ or exp Social Media/ or exp Human Computer Interaction/ or exp Telemedicine/ or Digital Interventions/ or Electronic Health Services/ or Mobile Health/ or Wearable Devices/ or Games/ or Computer Games/ or Computer Applications/ or Digital Gaming/ or Smartphones/ or Virtual Reality/ or Virtual Reality Exposure Therapy/ or exp Artificial Intelligence/ or ((tele adj (health or med* or psychology or psychiatry or therap* or care or conferenc* or communicat* or consult*)) or telemed* or telepsychology or telepsychiatry or teletherap* or telecare or teleconference* or telecommunicat* or teleconsult* or (social adj (medium? or media? or network* or platform?)) or telehealth or ("e" adj (health or treat* or therap* or counsel* or mail* or support*)) or ehealth or etreat* or etherap* or ecounsel* or email* or support* or m-health or mhealth or ((internet or online or "on line" or cyber or web) adj (based* or treat* or counsel* or therap* or prevention*)) or webcam* or "web cam*" or internetbased or onlinebased or cyberbased or cybercounsel* or video* or skyp* or computer* or electronic* or digital* or technolog* or robot* or telerobotics or audio* or helpline? or ((help or support) adj line?) or ((distance or remote or mobile) adj (counsel* or health)) or text-messag* or textmessag* or messaging or SMS or texting* or short message service? or mobile? or ((smart or cell*) adj phone?) or smartphone? or cellphone? or telephon* or blended* or app or apps or ((handheld or "hand held") adj device?) or iPad* or iPhone* or "i-pad*" or "i-phone*" or tablet* or sensor* or wearable* or palmtop? or "palm top?" or WhatsApp or "Whats App" or Twitter or tweet? or Facebook or facetim* or Instagram or Snapchat or TikTok or "Tik Tok" or WeChat or forum or chat* or (virtual adj (realit* or coach* or character? or human? or assist* or system? or medicine)) or VR or avatar? or ((conversation* or embodied or relational or interactive or virtual) adj agent?) or (serious adj (game? or gaming)) or gamification or ((ICT or "e" or internet or online or "on line" or web) adj intervention?) or "web 2.0*" or "web 3.0*" or wireless or world wide web or AI or "artificial intelligence").tw. | 1471040 |
| 3 | 1 and 2 | 14649 |
| 4 | 2025*.yr,dp,up. | 17037 |
| 5 | (202409* or 202410* or 202411* or 202412*).up. | 65177 |
| 6 | 3 and (4 or 5) | 593 |

**Database:** Sociological Abstracts via ProQuest

**Date:** 03 February 2025

**Number of hits:** 109

| S1 | SU.EXACT("Loneliness" or "Social Isolation") | 8,185 |
| --- | --- | --- |
| S2 | [STRICT] AB,TI(loneliness or lonely or (social* P/0 (exclusion or exclusions or excluded or isolation or isolated))) | 16,539 |
| S3 | [S1] OR [S2] | 18,413 |
| S4 | SU.EXACT("Computers" or "Mobile phones" or "Human-computer interaction" or "Social media" or "Virtual reality" or "Artificial intelligence") | 17,065 |
| S5 | [STRICT] AB,TI((tele P/0 (health OR med* OR psychology OR psychiatry OR therap* OR care OR conferenc* OR communicat* OR consult*)) OR telehealth OR telemed* OR telepsychology OR telepsychiatry OR teletherap* OR telecare OR teleconference* OR telecommunicat* OR teleconsult* OR (social P/0 (medium OR mediums OR media OR medias OR network* OR platform OR platforms)) OR ("e" P/0 (health OR treat* OR therap* OR counsel* OR mail* OR support*)) OR ehealth OR etreat* or etherap* OR ecounsel* OR email* OR support* OR "m-health" OR mhealth OR ((internet OR online OR "on line" OR cyber OR web) P/0 (based* OR treat* OR counsel* OR therap* OR prevention*)) OR webcam* OR "web cam*" OR internetbased OR onlinebased OR cyberbased OR cybercounsel* OR video* OR skyp* OR computer* OR electronic* OR digital* OR technolog* OR robot* OR telerobotics OR audio* OR helpline* OR ((help OR support) P/0 line*) OR ((distance OR remote OR mobile) P/0 (counsel* OR health)) OR "text-messag*" OR textmessag* OR messaging OR SMS OR texting* OR "short message service*" OR mobile OR mobiles OR ((smart OR cell*) P/0 phone*) OR smartphone* OR cellphone* OR telephon* OR blended* OR app OR apps OR ((handheld OR "hand held") P/0 device*) OR iPad* or iPhone* or "i-pad*" or "i-phone*" OR tablet* OR sensor* OR wearable* OR palmtop* OR "palm top*" OR WhatsApp or "Whats App" OR Twitter OR tweet OR tweets OR Facebook OR facetim* OR Instagram OR Snapchat or TikTok or "Tik Tok" or WeChat OR forum OR chat* OR (virtual P/0 (realit* OR coach* OR character OR characters OR human OR humans OR assist* OR system OR systems OR medicine)) OR VR OR avatar* OR ((conversation* OR embodied OR relational OR interactive OR virtual) P/0 (agent OR agents)) OR (serious P/0 (game OR games OR gaming)) OR gamification OR ((ICT OR "e" OR internet OR online OR "on line" OR web) P/0 intervention*) OR "web 2.0*" OR "web 3.0*" OR wireless OR "world wide web" OR AI OR "artificial intelligence") | 494,319 |
| S6 | [S4] OR [S5] | 495,727 |
| S7 | [S3] AND [S6] | 6,682 |
| S8 | [S3] AND [S6]  Limits applied: Publication date 2024-10-25 to 2025-02-03 | 109 |

**Database: Web of Science Core Collection**

**Science Citation Index Expanded**

**(SCI-EXPANDED)--1987-present**

**Social Sciences Citation Index**

**(SSCI)--1987-present**

**Arts & Humanities Citation Index**

**(AHCI)--1987-present**

**Emerging Sources Citation Index**

**(ESCI)—2019-present**

**Date:** 03 February 2025

**Number of hits:** 728

| 1 | TS=(loneliness or lonely or (social* NEAR/0 (exclusion$ or excluded or isolation or isolated))) | exact search | 56,763 |
| --- | --- | --- | --- |
| 2 | TS=((tele NEAR/0 (health or med* or psychology or psychiatry or therap* or care or conferenc* or communicat* or consult*)) or telehealth or telemed* or telepsychology or telepsychiatry or teletherap* or telecare or teleconference* or telecommunicat* or teleconsult* or (social NEAR/0 (medium$ or media$ or network* or platform$)) or ("e" NEAR/0 (health or treat* or therap* or counsel* or mail* or support*)) or ehealth or etreat* or etherap* or ecounsel* or email* or support* or "m-health" or mhealth or ((internet or online or "on line" or cyber or web) NEAR/0 (based* or treat* or counsel* or therap* or prevention*)) or webcam* or "web cam*" or internetbased or onlinebased or cyberbased or cybercounsel* or video* or skyp* or computer* or electronic* or digital* or technolog* or robot* or telerobotics or audio* or helpline$ or ((help or support) NEAR/0 line$) or ((distance or remote or mobile) NEAR/0 (counsel* or health)) or "text-messag*" or textmessag* or messaging or SMS or texting* or "short message service$" or mobile$ or ((smart or cell*) NEAR/0 phone$) or smartphone$ or cellphone$ or telephon* or blended* or app or apps or ((handheld or "hand held") NEAR/0 device$) or iPad* or iPhone* or "i-pad*" or "i-phone*" or tablet* or sensor* or wearable* or palmtop$ or "palm top$" or WhatsApp or "Whats App" or Twitter or tweet$ or Facebook or facetim* or Instagram or Snapchat or TikTok or "Tik Tok" or WeChat or forum or chat* or (virtual NEAR/0 (realit* or coach* or character$ or human$ or assist* or system$ or medicine)) or VR or avatar$ or ((conversation* or embodied or relational or interactive or virtual) NEAR/0 agent$) or (serious NEAR/0 (game$ or gaming)) or gamification or ((ICT or "e" or internet or online or "on line" or web) NEAR/0 intervention$) or "web 2.0*" or "web 3.0*" or wireless or "world wide web" or AI or "artificial intelligence") | exact search | 9,826,602 |
| 3 | #1 AND #2 | exact search | 24,711 |
| 4 | #3  Timespan: 2024-10-25 to 2025-02-03 | exact search | 728 |

**Database: Cinahl via Ebsco**

**Date:** 03 February 2025

**Number of hits:** 431

((MH "Loneliness") or (MH "Social Isolation") OR TI(loneliness or lonely or (social* W0 (exclusion# or excluded or isolation or isolated))) OR AB (loneliness or lonely or (social* W0 (exclusion# or excluded or isolation or isolated)))) AND ((MH "Computers, Portable") or (MH "Computers and Computerization") or (MH "Computers, Hand-Held") or (MH "User-Computer Interface") or (MH "Mobile Applications") or (MH "Social Media") or (MH "Twitter") or (MH "Telemedicine") or (MH "Telerehabilitation") or (MH "Telehealth") or (MH "Telepsychiatry") or (MH "Robotics") or (MH "Virtual Reality") or (MH "Virtual Reality Exposure Therapy") or (MH "Smartphone") or (MH "Text Messaging") or (MH "Cellular Phone") or (MH "Artificial Intelligence") OR TI((tele W0 (health or med* or psychology or psychiatry or therap* or care or conferenc* or communicat* or consult*)) OR telehealth OR telemed* or telepsychology OR telepsychiatry OR teletherap* OR telecare OR teleconference* OR telecommunicat* OR teleconsult* or (social W0 (medium# or media# or network* or platform#)) OR ("e" W0 (health or treat* or therap* or counsel* or mail* or support*)) or ehealth OR etreat* or etherap* or ecounsel* OR email* OR support* OR "m-health" OR mhealth OR ((internet or online or "on line" or cyber or web) W0 (based* or treat* or counsel* or therap* or prevention*)) or webcam* or "web cam*" or internetbased or onlinebased or cyberbased OR cybercounsel* or video* or skyp* or computer* OR electronic* OR digital* OR technolog* OR robot* or telerobotics or audio* or helpline# or ((help or support) W0 line#) or ((distance or remote or mobile) W0 (counsel* or health)) OR "text-messag*" OR textmessag* or messaging OR SMS OR texting* OR "short message service#" OR mobile# OR ((smart or cell*) W0 phone#) OR smartphone# or cellphone# OR telephon* or blended* OR app OR apps OR ((handheld or "hand held") W0 device#) OR iPad* or iPhone* or "i-pad*" or "i-phone*" OR tablet* OR sensor* OR wearable* OR palmtop# OR "palm top#" OR WhatsApp or "Whats App" OR Twitter or tweet# OR Facebook or facetim* OR Instagram OR Snapchat or TikTok or "Tik Tok" or WeChat OR forum OR chat* OR (virtual W0 (realit* or coach* or character# or human# or assist* or system# or medicine)) OR VR OR avatar# OR ((conversation* or embodied or relational or interactive or virtual) W0 agent#) OR (serious W0 (game# OR gaming)) OR gamification OR ((ICT or "e" or internet or online or "on line" or web) W0 intervention#) or "web 2.0*" or "web 3.0*" or wireless or "world wide web" or AI or "artificial intelligence") OR AB((tele W0 (health or med* or psychology or psychiatry or therap* or care or conferenc* or communicat* or consult*)) OR telehealth OR telemed* or telepsychology OR telepsychiatry OR teletherap* OR telecare OR teleconference* OR telecommunicat* OR teleconsult* or (social W0 (medium# or media# or network* or platform#)) OR ("e" W0 (health or treat* or therap* or counsel* or mail* or support*)) or ehealth OR etreat* or etherap* or ecounsel* OR email* OR support* OR "m-health" OR mhealth OR ((internet or online or "on line" or cyber or web) W0 (based* or treat* or counsel* or therap* or prevention*)) or webcam* or "web cam*" or internetbased or onlinebased or cyberbased OR cybercounsel* or video* or skyp* or computer* OR electronic* OR digital* OR technolog* OR robot* or telerobotics or audio* or helpline# or ((help or support) W0 line#) or ((distance or remote or mobile) W0 (counsel* or health)) OR "text-messag*" OR textmessag* or messaging OR SMS OR texting* OR "short message service#" OR mobile# OR ((smart or cell*) W0 phone#) OR smartphone# or cellphone# OR telephon* or blended* OR app OR apps OR ((handheld or "hand held") W0 device#) OR iPad* or iPhone* or "i-pad*" or "i-phone*" OR tablet* OR sensor* OR wearable* OR palmtop# OR "palm top#" OR WhatsApp or "Whats App" OR Twitter or tweet# OR Facebook or facetim* OR Instagram OR Snapchat or TikTok or "Tik Tok" or WeChat OR forum OR chat* OR (virtual W0 (realit* or coach* or character# or human# or assist* or system# or medicine)) OR VR OR avatar# OR ((conversation* or embodied or relational or interactive or virtual) W0 agent#) OR (serious W0 (game# OR gaming)) OR gamification OR ((ICT or "e" or internet or online or "on line" or web) W0 intervention#) or "web 2.0*" or "web 3.0*" or wireless or "world wide web" or AI or "artificial intelligence"))

Active filters:

- Publication date: 09/25/2024 - 02/03/2025
- Exclude MEDLINE records

**Database:** Cochrane Central Register of Controlled Trials

Issue 12 of 12, December 2024

**Date:** 04 February, 2025

**Number of hits:** 120

| #1 | [mh ^Loneliness] or [mh ^"Social Isolation"] | 532 |
| --- | --- | --- |
| #2 | (loneliness or lonely or (social* NEXT (exclusion? or excluded or isolation or isolated))):ti,ab | 2743 |
| #3 | #1 or #2 | 2910 |
| #4 | [mh Computers] or [mh "Computers, Handheld"] or [mh ^"Computer User Training"] or [mh ^"Mobile Applications"] or [mh ^"Social Media"] or [mh "User-Computer Interface"] or [mh ^Telemedicine] or [mh ^"Distance Counseling"] or [mh ^Robotics] or [mh ^"Wearable Electronic Devices"] or [mh ^Gamification] or [mh "Virtual Reality"] or [mh ^"Internet-Based Intervention"] or [mh "Artificial Intelligence"] | 16016 |
| #5 | ((tele NEXT (health or med* or psychology or psychiatry or therap* or care or conferenc* or communicat* or consult*)) OR telehealth OR telemed* or telepsychology OR telepsychiatry OR teletherap* OR telecare OR teleconference* OR telecommunicat* OR teleconsult* or (social NEXT (medium? or media? or network* or platform?)) OR ("e" NEXT (health or treat* or therap* or counsel* or mail* or support*)) or ehealth OR etreat* or etherap* or ecounsel* OR email* OR support* OR "m-health" OR mhealth OR ((internet or online or "on line" or cyber or web) NEXT (based* or treat* or counsel* or therap* or prevention*)) or webcam* or (web NEXT cam*) or internetbased or onlinebased or cyberbased OR cybercounsel* or video* or skyp* or computer* OR electronic* OR digital* OR technolog* OR robot* or telerobotics or audio* or helpline? or ((help or support) NEXT line?) or ((distance or remote or mobile) NEXT (counsel* or health)) OR (text NEXT messag*) OR textmessag* or messaging OR SMS OR texting* OR ("short message" NEXT service?) OR mobile? OR ((smart or cell*) NEXT phone?) OR smartphone? or cellphone? OR telephon* or blended* OR app OR apps OR ((handheld or "hand held") NEXT device?) OR iPad* or iPhone* or ("i" NEXT (pad* or phone*)) OR tablet* OR sensor* OR wearable* OR palmtop? or (palm NEXT top?) OR WhatsApp or "Whats App" OR Twitter or tweet? OR Facebook or facetim* OR Instagram OR Snapchat OR TikTok OR "Tik Tok" OR WeChat OR forum OR chat* OR (virtual NEXT (realit* or coach* or character? or human? or assist* or system? or medicine)) OR VR OR avatar? OR ((conversation* or embodied or relational or interactive or virtual) NEXT agent?) OR (serious NEXT (game? OR gaming)) OR gamification OR ((ICT or "e" or internet or online or "on line" or web) NEXT intervention?) or (web NEXT ("2.0" or "3.0")) or wireless or "world wide web" or AI or "artificial intelligence"):ti,ab | 456324 |
| #6 | #4 or #5 | 458321 |
| #7 | #3 and #6 | 1796 |
| #8 | #7 with Cochrane Library publication date Between Sep 2024 and Feb 2025, in Trials | 120 |
| #9 | #7 with Publication Year from 2025 to 2025, in Trials | 0 |
| #10 | #8 or #9 | 120 |

Loneliness and social isolation

Effect of digital interventions against loneliness and social isolation

-updated search

**Contact person:** Thomas Hansen

**Search:**  Ragnhild Agathe Tornes

**Date:** 3 February 2025

**Number of matches before duplicate control:** 27

**Number of matches after duplicate control:** 391 (in addition, look at links for hits that could not be exported)

| DATABASE | DATE | SEARCH | HITS |
| --- | --- | --- | --- |
| Swemed+  <https://svemedplus.kib.ki.se/> | -- | \| Hasn't been updated since 2019, so nothing to pick up here. \|  \| \| --- \| --- \| | --- |
| ClinicalTrials.gov  [www.clinicaltrials.gov](http://www.clinicaltrials.gov) | 3 February 2025 | Loneliness  First posted: From 10/09/2024 to 02/03/2025  Social isolation  First posted: From 10/09/2024 to 02/03/2025 | 12  8 |
| Open Grey System for Information on Grey Literature in Europe  <https://lifesciences.datastations.nl/dataverse/root/search> | -- | Changed search inerface, could not perform the same serach as the original search | --- |
| SBU – Statens beredning för medicinsk och social utvärdering  <https://www.sbu.se/sv/> | 3 February 2025 | The results cannot be exported. Some new hits after the original search. Browse through hitlists (choose "Sortera efter: Datum" to get newest first):  ensamhet: <https://www.sbu.se/sv/sok/?q=ensamhet&p=1&s=1&ps=10>  social isolering: <https://www.sbu.se/sv/sok/?q=social%20isolering&p=1&s=0&ps=10> | see links |
| WHO Library Catalog  <https://kohahq.searo.who.int/cgi-bin/koha/opac-search.pl> | 3 February 2025 | Advanced search:  Keyword: loneliness  Keyword: social isolation  Publication date range: 2024-2025 | 0 |
| WHO IRIS  <http://apps.who.int/iris/> | 9 October 2024 | Browsing by Subject "Loneliness"  Browsing by Subject "Social isolation" | 1  (from 2024-2025)  0  (from 2024-2025) |
| [Mednar](https://mednar.com/mednar/desktop/en/search.html)  <https://mednar.com/mednar/desktop/en/search.html> | 3 February 2025 | Advanced search: Full Record: loneliness / From: 2024 / To: 2025  Limited to Medical  (nothing new on social isolation) | 6 |
| [Socialstyrelsen](https://socialstyrelsen.dk/udgivelser) | 3 February 2025 | Search: ensamhet  175 resultat med avgrensinga:  Go to: "Dokument och filer” and limit to Innholdstyp: Publikationer  Sort on: “Datum" and browse through 2024-2025:  <https://www.socialstyrelsen.se/sok/?q=ensamhet> | See link |
| [Statens Institut for Folkesundhed](https://www.sdu.dk/da/sif/rapporter) | 3 February 2025 | Search: ensomhed  99 hits  Same number as the original search. Conclusion: tnothing new has been added here |  |

**APPENDIX 2**

Table. Systematic reviews of the efficacy of interventions to reduce social isolation and/or loneliness.

| Author/  year | Type of review | Aim | Population | Interventions | Study design | Out-come | Findings regarding digital interventions |
| --- | --- | --- | --- | --- | --- | --- | --- |
| Ambagt-sheer 2024 | SR | To identify theoretical perspectives, assess intervention effectiveness, and identify barriers and enablers of technology-based interventions targeting [social isolation](https://www.sciencedirect.com/topics/social-sciences/social-alienation) and loneliness in community-dwelling older adults. | Older adults | Digital** | Mixed | SI/L | Analyzing 19 studies, this SR found limited theoretical grounding in intervention designs and significant heterogeneity in approaches. Many interventions showed promise, with some reducing loneliness or increasing social networks, though findings were inconsistent due to varying study designs and small sample sizes. Barriers included resource demands, participant health, literacy, and technical challenges, while enablers involved tailoring to needs, engaging trainers, and fostering digital literacy. Future efforts should focus on scalable, theory-driven interventions addressing diverse needs in aging populations, considering both technological and non-technological solutions. |
| Balki 2022 | UR | To identify, synthesize, and critically appraise the effectiveness of technology interventions improving social connectedness in older adults | Older adults | Digital** | Mixed | SI/L | The reviews covered 326 primary studies with 79,538 participants.  The included reviews were dedicated to information and communications technology (ICT; 11/24, 46%), videoconferencing (4/24, 17%), computer or internet training (3/24, 12%), telecare (2/24, 8%), social networking sites (2/24, 8%), and robotics (2/27, 8%). Although technology was found to improve social connectedness, its effectiveness depended on study design and is improved by shorter durations, longer training times, and the facilitation of existing relationships. ICT and videoconferencing showed the best results, followed by computer training. Social networking sites achieved mixed results. Robotics and augmented reality showed promising results but lacked sufficient data for informed conclusions. The overall quality of the studies based on GRADE was medium low to very low. The lack of randomized controlled trials in underlying primary studies (<28%) and suboptimal methodologies limited our findings. |
| Beckers 2020* | UR |  | General | All | Mixed | SI/L | The review examined the effectiveness of interventions to tackle loneliness through 37 systematic reviews and meta-analyses. Interventions, including cognitive training, social skills enhancement, social support, and community-based approaches, were generally effective but varied in impact based on target groups and methods. Technology-based interventions showed mixed results, with cultural and individual factors influencing outcomes. Animal-assisted, physical activity, and place-based interventions demonstrated potential, though evidence was limited. Research gaps include the need for interventions tailored to youth, individuals with disabilities, and community-level strategies. Future studies should emphasize robust methodologies, long-term outcomes, and inclusivity in intervention design for diverse populations. |
| Boulton 2020* | UR |  | Older adults | Digital | Mixed | SI/L | Video-communication interventions may decrease loneliness and social isolation. Telephone befriending, online discussion groups and forums, social networking sites, and multi-tool interventions may lead to little or no difference in decreasing loneliness and social isolation. Effect sizes and heterogeneity of effect sizes are not discussed. |
| Chipps 2017 | UR | To synthesize high quality evidence on the effectiveness of e-Interventions to decrease social isolation/loneliness for older people living in community/residential care | Older adults | Digital | Mixed (RCTs and non-RCTs) | SI/L | The final search identified 12 reviews, which included 22 unique primary research studies evaluating e-Interventions for social isolation or loneliness. The reviews were of moderate quality and the primary studies showed a lack of rigor. There was little or no difference for digital interventions so it is uncertain whether digital interventions (e.g., online activities, internet-supported communication, internet/computer training) decrease loneliness. |
| Chua 2024 | SR/MA | To evaluate the effectiveness of home-based interventions in improving loneliness and social connectedness (primary outcomes), and depressive symptoms (secondary outcome) among older adults | Older | Home-based | RCTs | SI/L | 14 RCTs were included (all published before 2020). Home-based interventions were found to significantly increase older adults’ social connectedness (social support and social engagement) and reduce their loneliness and depressive symptoms. Subgroup analyses suggested that interventions which lasted more than three months and were delivered using mixed platforms were more favorable. Both professional-led and volunteer-led interventions showed favorable results. |
| Duffner 2024 | SR/MA | To summarize all available evidence regarding the effectiveness of interventions for loneliness and social isolation, to map out their working mechanisms, and to give implications for policy and practice. | Older adults | All | Mixed | SI/L | The review analyzed 67 studies on interventions targeting loneliness and social isolation in older adults. Meta-analysis of 27 studies revealed a medium overall effect size (Cohen’s d = -0.47) for loneliness interventions, with 63% reporting sustained effects during follow-up. Effective interventions clustered into three mechanisms: promoting social contact, transferring knowledge/skills, and addressing social cognition. Non-technology-based interventions showed slightly better effectiveness. Despite heterogeneity and limitations, findings underline the potential of person-centered, multifaceted approaches tailored to individual needs. Future research should emphasize long-term outcomes, cost-effectiveness, and multidimensional frameworks for understanding loneliness and guiding targeted interventions for diverse populations.  10 studies (614 participants in total) that are technological-based. The meta-analysis shows benefits (d= -0.47 [-0.62; -0.32]). |
| Döring 2024 | Scoping | To summarize the communication technologies (CTs), theoretical frameworks, study designs, and positive effects of technology use present in the research field | Older adults | Digital | Mixed | SI/L | The majority of the included reviews addressed general internet and computer use (82% each). Of the 28 reviews, only one (4%) worked with a theoretical framework and 26 (93%) covered primary studies with quantitative-experimental designs. The positive effects of technology use were shown in 55% of the outcome measures for loneliness and 44% of the outcome measures for social isolation. Conclusion: While research reviews show that CTs can reduce loneliness and social isolation in older people, causal evidence is limited. |
| Hansen 2022 | UR | To evaluate the effects of all types of interventions, based on RCT-based evidence from systematic reviews. | All ages | All | RCTs | SI/L | The umbrella review highlighted the mixed effectiveness of digital interventions for reducing loneliness and social isolation. These interventions often included online support groups, video calls, and technology training. Meta-analyses suggested small-to-moderate beneficial effects, though findings were inconsistent due to heterogeneous intervention designs and participant characteristics. |
| Hao 2023 | SR/MA | To evaluate the effects of telehealth interventions on HRQoL and psychological outcomes including anxiety, depression, perceived stress, resilience, loneliness, well-being, and self-efficiency in community adults during the COVID-19 pandemic | All ages | Telehealth | Mixed | L | The interventions were conducted via smartphone, videoconferencing platform, telephone, social networking, or other telehealth technologies. Only 4 RCTs on loneliness (Brog 2022, Gilbody 2021, Kahlon 2021, Shapira 2021). The pooled results of four studies did not show a significant effect on loneliness level (SMD = −0.63, 95% CI: −1.44, 0.18, I2 =93.9%, p = 0.129). Two studies showed significantly positive effects of telehealth on loneliness, both explored interventions delivered by videoconferencing or telephone. |
| Jarvis 2020 | UR | To evaluate the effects of digital interventions targeting loneliness among older adults. | Older adults | All | Mixed | L | No evidence of effect on loneliness irrespective of delivery format (digital or non-digital) or intervention type (social support, social contact, social skills training). |
| Li 2023 | SR/MA | To examine the effects of digital interventions aimed at reducing loneliness among older adults during medical pandemics, especially COVID-19 | Older | Psycho-logical | Mix | L | It analyzed 12 studies, finding that web-based solutions like virtual support groups, cognitive training, and online mindfulness programs were the most commonly employed. While single-modal digital interventions showed moderate success in reducing loneliness, their effects were often short-term. Barriers included varying levels of digital literacy, technology access, and intervention scalability. Multi-modal approaches yielded mixed outcomes, emphasizing the need for tailored, user-centric designs. Future efforts should focus on sustainable, inclusive, and scalable digital solutions to address the unique challenges of older populations during crises​.  4 RCTs, mixed effects. Studies from 2021 and 2022. |
| Nichol 2024 | UR | To explore the effect of socially assistive robots within health and social care on psychosocial, behavioural, and wellbeing outcomes across the lifespan | General | Socially assistive robots | Mix | L | Socially assistive robots show promise for improving non-psychiatric outcomes such as loneliness, positive affect, stress, and pain, but exert no effect on psychiatric outcomes such as depression and agitation. |
| Pallavicini 2022 | SR | To describe the literature on the effects of video games during the early stages of the COVID-19 crisis on stress, anxiety, depression, loneliness, and gaming disorder (GD) | General | Video games | Mixed | L | The systematic review explored the role of video games during the early stages of the COVID-19 pandemic in alleviating stress, anxiety, depression, and loneliness. The review found that online multiplayer and augmented reality games provided social connectivity and emotional relief for many individuals, especially young adults. For some, gaming reduced feelings of isolation and enhanced well-being. However, excessive gaming was associated with negative outcomes, such as increased loneliness and gaming disorder symptoms, particularly in at-risk populations. The findings emphasize the dual potential of video games as a coping tool and a risk factor, contingent on individual circumstances and usage patterns​. No RCTs on loneliness. |
| Plackett 2023 | SR | To synthesize the literature on the effectiveness of social media use interventions in improving mental well-being in adults. | General | Social media abstenence/limitation | RCTs | L | The systematic review examined the effectiveness of social media use interventions on improving mental well-being. Therapy-based approaches, including cognitive behavioral therapy and mindfulness training, were most effective, with 83% of such studies showing positive outcomes like reduced depression and loneliness. Limiting social media use or full abstinence showed mixed results, with many studies finding no significant improvements or even negative effects, such as increased loneliness. Barriers included low adherence rates and limited sustainability. The review highlights the need for personalized, reflective interventions rather than mere usage reduction, emphasizing therapy-based methods for better long-term mental well-being outcomes​  5 RCTs (Chen 2022, Hunt 2018, Hall 2019, Vanman 2018, Vally 2019). These focus on limiting or abstinence of SM use. 2/5 studies find effect. |
| Sha 2022 | SR/MA | To investigate the impact of relational agents on loneliness across age groups | General | Relational agents | Mix | L | The meta-analysis reviewed the effectiveness of relational agents—AI-powered entities like chatbots and social robots—on loneliness across various age groups. Including 14 studies with 286 participants, findings revealed a moderate reduction in loneliness (Hedge’s g = -0.552). Social robotic agents showed stronger effects compared to app-based agents. Key mechanisms included companionship and facilitating social interaction. Barriers included small study sizes, high attrition rates, and insufficient integration of behavioral theories. Relational agents hold promise as scalable loneliness interventions, especially when integrated into multi-component approaches tailored to user needs. Future research should focus on younger demographics, design optimization, and long-term efficacy.  5 RCTs (Banks 2008, Bickmore 2005, Robinson 2013, Papadopoulos 2022, Loveys 2021). Five studies were available for the RCT-only model. Hedge’s g was -0.437 (Z=-2.495; 95% CI, -0.781 to -0.094; P=0.013), which was 21% less than the estimate of the main model. The results were significant at a traditional α=0.05 but not at the αBonferroni. |
| Shekelle 2024 | SR/MA | To conduct a systematic review and meta-analysis of diverse interventions to reduce loneliness in older community-living adults, | Older | All | Mix | L | Internet training programs were moderately effective, with a standardized mean difference of -0.22 (95% CI -0.30, -0.14) in randomized controlled trials. These programs included teaching older adults basic computer and internet skills, social media use, and video communication. Internet-delivered interventions, such as online cognitive behavioral therapy, showed smaller but significant effects (standardized mean difference -0.27, 95% CI -0.53, -0.01). Effectiveness depended on engagement levels, with some barriers including digital literacy gaps and accessibility issues.  RCTs: Internet training: 5 RCT studies= −0.22 (95% confidence interval − 0.30, − 0.14. Internet-delivered: 7 RCTSs. SMD = −0.27 (95% CI−0.53,−0.01)). ES for the 3 RCTs of CBT were higher than for any of the other interventions. |
| Veronese 2021 | UR | To explore effects of various interventions to alleviate loneliness, including technological ones such as video calls and robopets. | General | All | RCTs | L | Digital interventions did not significantly reduce loneliness in the RCTs analyzed. Challenges included insufficient evidence, limited sample sizes, and high variability in outcomes. While some observational studies suggested potential benefits for older adults, especially during the COVID-19 pandemic, the review emphasized the need for more robust research to establish the efficacy of these interventions and improve their design to meet diverse user needs. |
| Victor 2018* | UR | To assessed interventions targeting loneliness across the life course, including digital solutions such as video calls and computer training. | General | All | RCTs | L | Evidence on digital interventions was mixed, with some studies indicating reduced loneliness through activities like videoconferencing with family or internet training. However, the impact varied, and some interventions reinforced isolation when participants lacked technological literacy or support. Challenges included heterogeneity in study design and limited data on long-term effects. Tailored, person-centered approaches addressing users' specific needs, particularly for vulnerable groups, were recommended as more effective in reducing loneliness through digital means​. |
| Yen 2024 | SR/MA | To explore the effects of concrete forms of social robots on depression and loneliness in older residents in long-term care facilities by a systematic review and meta-analysis of RCTs. | Older | Social robots | RCTs | L | 8 studies were selected for both qualitative and quantitative synthesis. Social robot interventions had significant positive effects on decreasing depression and loneliness with large effect sizes. All studies from before 2020. |

* Grey (non-peer reviewed) literature. ** Termed “technology interventions”
Abbrevations: SR = Systematic review, UR = Umbrella review (systematic review of systemativ reviews), SI = Social Isolation, L = Loneliness, RCT = Randomized controlled trial.

**APPENDIX 3**

**Table. Excluded studies, with reason for exclusion.**

| **Author** | **Reason for exclusion** |
| --- | --- |
| Tarver 2022 | Ongoing study (protocol, early stage, under review, etc.) |
| van Der Mei 2024 | Ongoing study (protocol, early stage, under review, etc.) |
| Natarajan 2022 | Ongoing study (protocol, early stage, under review, etc.) |
| Cooper 2025 | Ongoing study (protocol, early stage, under review, etc.) |
| Lydon 2024 | Ongoing study (protocol, early stage, under review, etc.) |
| Byun 2023 | Ongoing study (protocol, early stage, under review, etc.) |
| Higuchi 2023 | Ongoing study (protocol, early stage, under review, etc.) |
| Radin 2023 | Ongoing study (protocol, early stage, under review, etc.) |
| Walsh 2024 | ES data missing (e.g., N, mean, SD) |
| Fritz 2023 | ES data missing (e.g., N, mean, SD) |
| Haines 2024 | Ongoing study (protocol, early stage, under review, etc.) |
| Brodbeck 2022 | Ongoing study (protocol, early stage, under review, etc.) |
| King 2023 | Ongoing study (protocol, early stage, under review, etc.) |
| University of Colorado 2024 | Ongoing study (protocol, early stage, under review, etc.) |
| Kwan 2023 | Ongoing study (protocol, early stage, under review, etc.) |
| Simes 2022 | Ongoing study (protocol, early stage, under review, etc.) |
| Janssen Jeroen 2023 | Wrong outcomes |
| Muneton-CastanoYudy 2022 | Wrong outcomes |
| Tu 2023 | Ongoing study (protocol, early stage, under review, etc.) |
| Kennedy 2025 | Ongoing study (protocol, early stage, under review, etc.) |
| Schueller 2024 | Ongoing study (protocol, early stage, under review, etc.) |
| Achtert 2025 | Ongoing study (protocol, early stage, under review, etc.) |
| SeewerNoemi 2022 | Ongoing study (protocol, early stage, under review, etc.) |
| Norberg 2024 | Ongoing study (protocol, early stage, under review, etc.) |
| ByunJun 2024 | Ongoing study (protocol, early stage, under review, etc.) |
| Qiyuan 2024 | Ongoing study (protocol, early stage, under review, etc.) |
| SoaresErin 2022 | Wrong control group (e.g., digital vs. digital) |
| O'RourkeH 2022 | Conference paper |
| KleinauE 2023 | Duplicate |
| Jung 2024 | ES data missing (e.g., N, mean, SD) |
| Shorey 2018 | Ongoing study (protocol, early stage, under review, etc.) |
| Han 2020 | Ongoing study (protocol, early stage, under review, etc.) |
| Grudzen 2024 | Conference paper |
| Poppe 2022 | Ongoing study (protocol, early stage, under review, etc.) |
| Gustafson 2022 | Ongoing study (protocol, early stage, under review, etc.) |
| Burke 2022 | Ongoing study (protocol, early stage, under review, etc.) |
| Mccormick 2024 | Ongoing study (protocol, early stage, under review, etc.) |
| Schmitt 2024 | Ongoing study (protocol, early stage, under review, etc.) |
| Xiang 2024 | Ongoing study (protocol, early stage, under review, etc.) |
| ZhengDavid 2024 | Ongoing study (protocol, early stage, under review, etc.) |
| Davis 2023 | Ongoing study (protocol, early stage, under review, etc.) |
| Eliacin 2024 | Ongoing study (protocol, early stage, under review, etc.) |
| Yan 2024 | Ongoing study (protocol, early stage, under review, etc.) |
| OfosuE 2023 | No RCT |
| Crawford 2024 | Ongoing study (protocol, early stage, under review, etc.) |
| Gray 2024 | Ongoing study (protocol, early stage, under review, etc.) |
| Ekitli 2023 | Ongoing study (protocol, early stage, under review, etc.) |
| Osorno 2024 | Conference paper |
| YangShang-Yu 2023 | Wrong control group (e.g., digital vs. digital) |
| ZagicDino 2024 | ES data missing (e.g., N, mean, SD) |
| KallAnton 2023 | Main results published pre-2022 |
| Domenech 2024 | Ongoing study (protocol, early stage, under review, etc.) |
| Ashrafioun 2022 | Ongoing study (protocol, early stage, under review, etc.) |
| Leung 2022 | Ongoing study (protocol, early stage, under review, etc.) |
| Park 2022 | Ongoing study (protocol, early stage, under review, etc.) |
| Vonderlin 2023 | Ongoing study (protocol, early stage, under review, etc.) |
| Cipolletta 2023 | Ongoing study (protocol, early stage, under review, etc.) |
| Martines 2022 | Ongoing study (protocol, early stage, under review, etc.) |
| LimMichelle 2023 | Ongoing study (protocol, early stage, under review, etc.) |
| Li 2024 | Ongoing study (protocol, early stage, under review, etc.) |
| HenwoodBenjamin 2024 | Ongoing study (protocol, early stage, under review, etc.) |
| KleinauEckhard 2024 | Wrong control group (e.g., digital vs. digital) |
| DodgeHiroko 2024 | Wrong outcomes |
| JiangDa 2024 | Wrong control group (e.g., digital vs. digital) |
| Liu 2024 | Wrong control group (e.g., digital vs. digital) |
| ChanS 2024 | Ongoing study (protocol, early stage, under review, etc.) |
| Rej 2022 | Ongoing study (protocol, early stage, under review, etc.) |
| Matura 2024 | Ongoing study (protocol, early stage, under review, etc.) |
| Littlewood 2024 | Ongoing study (protocol, early stage, under review, etc.) |
| Donohoe 2024 | Ongoing study (protocol, early stage, under review, etc.) |
| Bellani 2023 | Ongoing study (protocol, early stage, under review, etc.) |
| Schick 2022 | Ongoing study (protocol, early stage, under review, etc.) |
| Oslo Metropolitan University 2023 | Ongoing study (protocol, early stage, under review, etc.) |
| KanerAvigdor 2024 | Wrong outcomes |
| RadinAnna 2023 | Wrong intervention (not digital) |
| Hussain 2023 | Wrong control group (e.g., digital vs. digital) |
| KwokJojoYanYan 2024 | Wrong control group (e.g., digital vs. digital) |
| ShafieiTouran 2024 | Ongoing study (protocol, early stage, under review, etc.) |
| KayserJay 2023 | Ongoing study (protocol, early stage, under review, etc.) |
| Hur 2022 | Ongoing study (protocol, early stage, under review, etc.) |
| Keisari 2024 | Ongoing study (protocol, early stage, under review, etc.) |
| YapKeong 2024 | Wrong control group (e.g., digital vs. digital) |
| McGillivrayLauren 2023 | Wrong control group (e.g., digital vs. digital) |
| Bystrom 2024 | Ongoing study (protocol, early stage, under review, etc.) |
| Lee 2023 | Ongoing study (protocol, early stage, under review, etc.) |
| WarnerLisa 2024 | Ongoing study (protocol, early stage, under review, etc.) |
| Hillegers 2022 | Ongoing study (protocol, early stage, under review, etc.) |
| Simon 2024 | Ongoing study (protocol, early stage, under review, etc.) |
| Yip 2024 | Ongoing study (protocol, early stage, under review, etc.) |
| Fainstad 2024 | ES data missing (e.g., N, mean, SD) |
| Cheung 2023 | Ongoing study (protocol, early stage, under review, etc.) |
| Shang-Yu 2022 | Ongoing study (protocol, early stage, under review, etc.) |
| Johnson 2023 | Conference paper |
| RyanMarian 2023 | Wrong intervention (not digital) |
| FunghiGiulia 2024 | Wrong control group (e.g., digital vs. digital) |
| AomoriMaki 2023 | ES data missing (e.g., N, mean, SD) |
| Chen 2024 | Ongoing study (protocol, early stage, under review, etc.) |
| GustafsonD 2024 | Wrong control group (e.g., digital vs. digital) |
| KangBoyoung 2024 | Wrong control group (e.g., digital vs. digital) |
| Brodbeck 2022 | Ongoing study (protocol, early stage, under review, etc.) |
| Franz 2022 | Ongoing study (protocol, early stage, under review, etc.) |
| Lee CK 2023 | Ongoing study (protocol, early stage, under review, etc.) |
| TangVivienFoongYee 2024 | Ongoing study (protocol, early stage, under review, etc.) |
| ShadeMarcia 2024 | Conference paper |
| Jiang 2025 | Wrong control group (e.g., digital vs. digital) |
| Albert 2024 | Ongoing study (protocol, early stage, under review, etc.) |
| Burke 2022 | Ongoing study (protocol, early stage, under review, etc.) |
| Löchner 2022 | Ongoing study (protocol, early stage, under review, etc.) |
| ScottJuliaE 2022 | Ongoing study (protocol, early stage, under review, etc.) |
| BartelsG 2024 | Record not found |
| Leung 2023 | Ongoing study (protocol, early stage, under review, etc.) |
| LavinPaola 2022 | Ongoing study (protocol, early stage, under review, etc.) |
| ArakawaYuki 2023 | ES data missing (e.g., N, mean, SD) |
| ChoiSeulKi 2022 | Wrong control group (e.g., digital vs. digital) |
| Boot 2023 | Ongoing study (protocol, early stage, under review, etc.) |
| Damberg2024 | Ongoing study (protocol, early stage, under review, etc.) |
| Gorentz 2022 | Ongoing study (protocol, early stage, under review, etc.) |
| RewLynnEdD 2022 | Wrong control group (e.g., digital vs. digital) |
| LiuChia-Wen 2023 | Wrong control group (e.g., digital vs. digital) |
| Michałowski 2023 | Ongoing study (protocol, early stage, under review, etc.) |
| Lee J 2023 | Ongoing study (protocol, early stage, under review, etc.) |
| Shin 2023 | Ongoing study (protocol, early stage, under review, etc.) |
| KatsukiFujika 2024 | Ongoing study (protocol, early stage, under review, etc.) |
| GrudzenCRR 2024 | Conference paper |
| TandonPuneeta 2022 | Ongoing study (protocol, early stage, under review, etc.) |
| BradwellHannah 2022 | Wrong outcomes |
| SeewerNoemi 2024 | ES data missing (e.g., N, mean, SD) |
| Stott 2024 | Ongoing study (protocol, early stage, under review, etc.) |
| LinXinYao 2023 | Wrong control group (e.g., digital vs. digital) |
| Samtani 2023 | Ongoing study (protocol, early stage, under review, etc.) |
| Newman 2022 | Ongoing study (protocol, early stage, under review, etc.) |
| Zhao 2024 | Ongoing study (protocol, early stage, under review, etc.) |
| NikpeymaNasrin 2024 | Ongoing study (protocol, early stage, under review, etc.) |
| MiuraKumiWatanabe 2023 | Ongoing study (protocol, early stage, under review, etc.) |
| Jeong 2024 | Conference paper |
| Heffner 2023 | Ongoing study (protocol, early stage, under review, etc.) |
| Xu 2023 | Ongoing study (protocol, early stage, under review, etc.) |
| Hessels 2022 | Ongoing study (protocol, early stage, under review, etc.) |
| ImaiAyu 2024 | Ongoing study (protocol, early stage, under review, etc.) |
| Lim 2023 | Ongoing study (protocol, early stage, under review, etc.) |
| Sloan 2023 | Ongoing study (protocol, early stage, under review, etc.) |
| Cooper 2024 | Ongoing study (protocol, early stage, under review, etc.) |
| Prochilo 2024 | Ongoing study (protocol, early stage, under review, etc.) |
| WongA 2024 | Wrong control group (e.g., digital vs. digital) |
| CzajaSara 2024 | Wrong control group (e.g., digital vs. digital) |
| YangMin 2024 | Ongoing study (protocol, early stage, under review, etc.) |
| BevilacquaRoberta 2024 | Ongoing study (protocol, early stage, under review, etc.) |
| Mo 2024 | Ongoing study (protocol, early stage, under review, etc.) |
| Kluger 2022 | Ongoing study (protocol, early stage, under review, etc.) |
| Schmidt 2022 | Ongoing study (protocol, early stage, under review, etc.) |
| Schipper-KramerFreher 2022 | Ongoing study (protocol, early stage, under review, etc.) |
| WestTaylor 2024 | Wrong control group (e.g., digital vs. digital) |
| IyerPriya 2024 | ES data missing (e.g., N, mean, SD) |
| Maradian 2022 | Ongoing study (protocol, early stage, under review, etc.) |
| Salman 2023 | Ongoing study (protocol, early stage, under review, etc.) |
| MastrogiovanniChiara 2022 | Ongoing study (protocol, early stage, under review, etc.) |
| KramerLean 2022 | ES data missing (e.g., N, mean, SD) |
| RiordanKevin 2024 | Wrong control group (e.g., digital vs. digital) |
| BinfetJ 2022 | Wrong control group (e.g., digital vs. digital) |
| ElkarifTalia 2024 | Wrong control group (e.g., digital vs. digital) |
| Yip 2024 | Ongoing study (protocol, early stage, under review, etc.) |
| Slavich 2023 | Ongoing study (protocol, early stage, under review, etc.) |
| KwanR 2023 | Ongoing study (protocol, early stage, under review, etc.) |
| BodensteinKatie 2024 | Ongoing study (protocol, early stage, under review, etc.) |
| Vonderlin 2022 | Ongoing study (protocol, early stage, under review, etc.) |
| WagnerBirgit 2022 | Ongoing study (protocol, early stage, under review, etc.) |
| Bordini 2022 | Ongoing study (protocol, early stage, under review, etc.) |
| Schöne 2023 | Ongoing study (protocol, early stage, under review, etc.) |
| AshrafiounLisham 2024 | Wrong control group (e.g., digital vs. digital) |
| ChoiT 2024 | Conference paper |

**APPENDIX 4. Risk of bias (RoB) assessments. Green = low RoB, yellow = moderate RoB, red = high RoB**

|  | Random sequence generation | Allocation concealment | Selective outcome reporting | Bliding of participants | Blinding of outcome assessment | Incomplete outcome data | Bias due to other problems | Overall |
| --- | --- | --- | --- | --- | --- | --- | --- | --- |
| Ae-Ri 2023 |  |  |  |  |  |  |  |  |
| Andrade 2023 |  |  |  |  |  |  |  |  |
| Berko 2023 |  |  |  |  |  |  |  |  |
| Boucher 2024 |  |  |  |  |  |  |  |  |
| Brog 2022 |  |  |  |  |  |  |  |  |
| Chen 2024 |  |  |  |  |  |  |  |  |
| Christie 2022 |  |  |  |  |  |  |  |  |
| de Hesselle 2024 |  |  |  |  |  |  |  |  |
| Demirag 2022 |  |  |  |  |  |  |  |  |
| DuPont 2023 |  |  |  |  |  |  |  |  |
| Dworschak 2024 |  |  |  |  |  |  |  |  |
| Faulhaber 2023 |  |  |  |  |  |  |  |  |
| Gilbody 2024 |  |  |  |  |  |  |  |  |
| Hirshberg 2024 |  |  |  |  |  |  |  |  |
| Iyer 2023 |  |  |  |  |  |  |  |  |
| Joosten 2024 |  |  |  |  |  |  |  |  |
| Kahlon 2023 |  |  |  |  |  |  |  |  |
| Karkosz 2024 |  |  |  |  |  |  |  |  |
| Lippke 2022 |  |  |  |  |  |  |  |  |
| Liu 2023 |  |  |  |  |  |  |  |  |
| Maj 2024 |  |  |  |  |  |  |  |  |
| Matthaeus 2024 |  |  |  |  |  |  |  |  |
| Muller 2024 |  |  |  |  |  |  |  |  |
| Mueller 2023 |  |  |  |  |  |  |  |  |
| Ozturk 2022 |  |  |  |  |  |  |  |  |
| Papadopoulos 2022 |  |  |  |  |  |  |  |  |
| Pena 2024 |  |  |  |  |  |  |  |  |
| Perkins 2023 |  |  |  |  |  |  |  |  |
| Purdie 2023 |  |  |  |  |  |  |  |  |
| Rubin 2024 |  |  |  |  |  |  |  |  |
| Seewer 2024 |  |  |  |  |  |  |  |  |
| Silva 2024 |  |  |  |  |  |  |  |  |
| Teo 2025 |  |  |  |  |  |  |  |  |
| Tuncgenc 2024 |  |  |  |  |  |  |  |  |
| Wagner 2024 |  |  |  |  |  |  |  |  |
| Walsh 2024a |  |  |  |  |  |  |  |  |
| Walsh 2024b |  |  |  |  |  |  |  |  |
| Warner 2024 |  |  |  |  |  |  |  |  |
| Wolgast 2023 |  |  |  |  |  |  |  |  |
| Xiang 2024 |  |  |  |  |  |  |  |  |

**APPENDIX 5. PRISMA Checklist**

| **Section/topic** | **#** | **Checklist item** | **Reported on page #** |
| --- | --- | --- | --- |
| **TITLE** | | |  |
| Title | 1 | Identify the report as a systematic review, meta-analysis, or both. | 1 |
| **ABSTRACT** | | |  |
| Structured summary | 2 | Provide a structured summary including, as applicable: background; objectives; data sources; study eligibility criteria, participants, and interventions; study appraisal and synthesis methods; results; limitations; conclusions and implications of key findings; systematic review registration number. | 1 |
| **INTRODUCTION** | | |  |
| Rationale | 3 | Describe the rationale for the review in the context of what is already known. | 2-4 |
| Objectives | 4 | Provide an explicit statement of questions being addressed with reference to participants, interventions, comparisons, outcomes, and study design (PICOS). | 4 |
| **METHODS** | | |  |
| Protocol and registration | 5 | Indicate if a review protocol exists, if and where it can be accessed (e.g., Web address), and, if available, provide registration information including registration number. | 5 |
| Eligibility criteria | 6 | Specify study characteristics (e.g., PICOS, length of follow-up) and report characteristics (e.g., years considered, language, publication status) used as criteria for eligibility, giving rationale. | 5-6 |
| Information sources | 7 | Describe all information sources (e.g., databases with dates of coverage, contact with study authors to identify additional studies) in the search and date last searched. | 6 |
| Search | 8 | Present full electronic search strategy for at least one database, including any limits used, such that it could be repeated. | Appendix 1 |
| Study selection | 9 | State the process for selecting studies (i.e., screening, eligibility, included in systematic review, and, if applicable, included in the meta-analysis). | 7 |
| Data collection process | 10 | Describe method of data extraction from reports (e.g., piloted forms, independently, in duplicate) and any processes for obtaining and confirming data from investigators. | 7-8 |
| Data items | 11 | List and define all variables for which data were sought (e.g., PICOS, funding sources) and any assumptions and simplifications made. | 6-7 |
| Risk of bias in individual studies | 12 | Describe methods used for assessing risk of bias of individual studies (including specification of whether this was done at the study or outcome level), and how this information is to be used in any data synthesis. | 8 and Appendix 4 |
| Summary measures | 13 | State the principal summary measures (e.g., risk ratio, difference in means). | 8-9 |
| Synthesis of results | 14 | Describe the methods of handling data and combining results of studies, if done, including measures of consistency (e.g., I^2^) for each meta-analysis. | 8-9 |
| Risk of bias across studies | 15 | Specify any assessment of risk of bias that may affect the cumulative evidence (e.g., publication bias, selective reporting within studies). | 9 |
| Additional analyses | 16 | Describe methods of additional analyses (e.g., sensitivity or subgroup analyses, meta-regression), if done, indicating which were pre-specified. | 9 |
| **RESULTS** | | |  |
| Study selection | 17 | Give numbers of studies screened, assessed for eligibility, and included in the review, with reasons for exclusions at each stage, ideally with a flow diagram. | 9 and figure 1 |
| Study characteristics | 18 | For each study, present characteristics for which data were extracted (e.g., study size, PICOS, follow-up period) and provide the citations. | Table 1 |
| Risk of bias within studies | 19 | Present data on risk of bias of each study and, if available, any outcome level assessment (see item 12). | Appendix 4 |
| Results of individual studies | 20 | For all outcomes considered (benefits or harms), present, for each study: (a) simple summary data for each intervention group (b) effect estimates and confidence intervals, ideally with a forest plot. | Figures 2-9 |
| Synthesis of results | 21 | Present results of each meta-analysis done, including confidence intervals and measures of consistency. | Figures 2-9 |
| Risk of bias across studies | 22 | Present results of any assessment of risk of bias across studies (see Item 15). | 10 and Appendix 4 |
| Additional analysis | 23 | Give results of additional analyses, if done (e.g., sensitivity or subgroup analyses, meta-regression [see Item 16]). | 11-14 |
| **DISCUSSION** | | |  |
| Summary of evidence | 24 | Summarize the main findings including the strength of evidence for each main outcome; consider their relevance to key groups (e.g., healthcare providers, users, and policy makers). | 14-20 |
| Limitations | 25 | Discuss limitations at study and outcome level (e.g., risk of bias), and at review-level (e.g., incomplete retrieval of identified research, reporting bias). | 14 and 18 |
| Conclusions | 26 | Provide a general interpretation of the results in the context of other evidence, and implications for future research. | 19 |
| **FUNDING** | | |  |
| Funding | 27 | Describe sources of funding for the systematic review and other support (e.g., supply of data); role of funders for the systematic review. | 20 |

**Appendix 6. Figures (funnel plot and forest plots).**

Figure A. Funnel plot. Meta-analysis of self-guided psychological interventions on loneliness (13 studies)


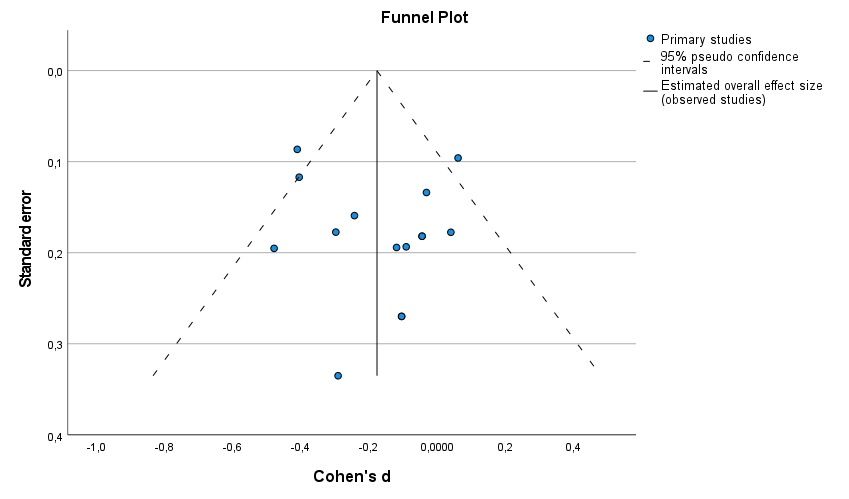


Figure B. Meta-analysis of self-guided psychological interventions on loneliness, after removing one outlier (Gilbody 2024) (13 studies)


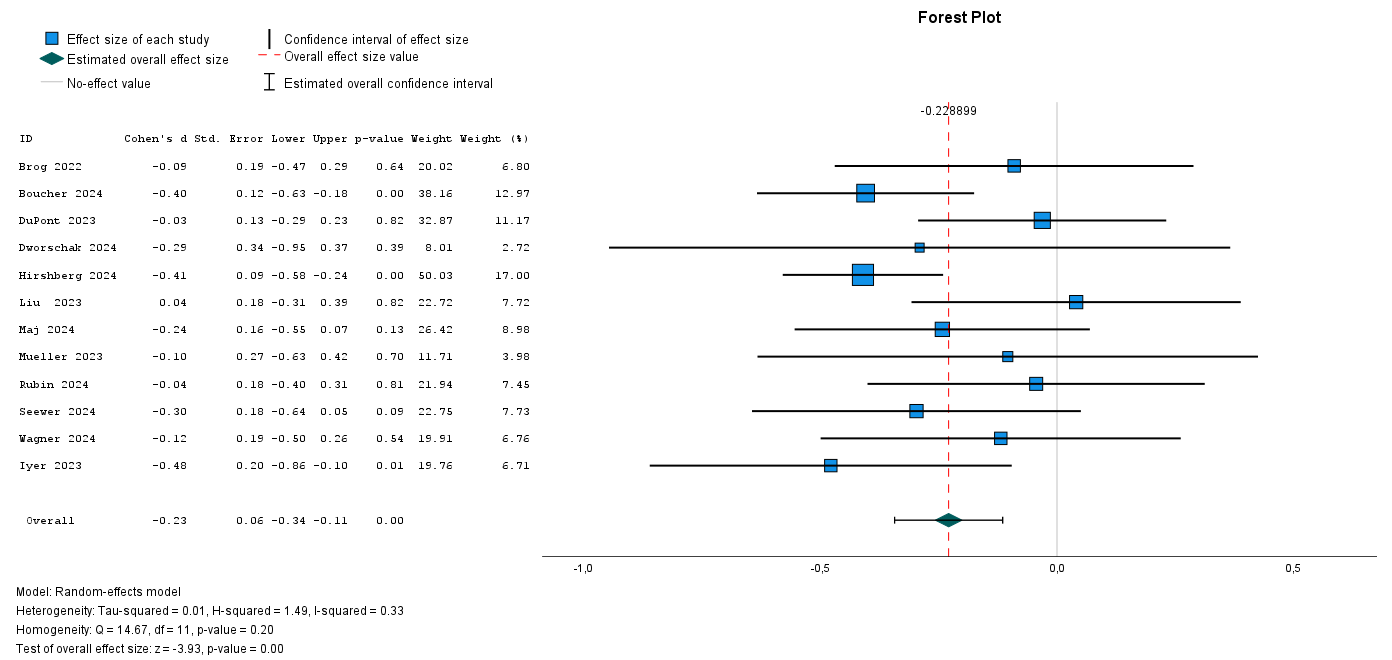


Figure C. Meta-analysis of self-guided psychological interventions on loneliness, including only studies with loneliness as a primary outcome (9 studies)


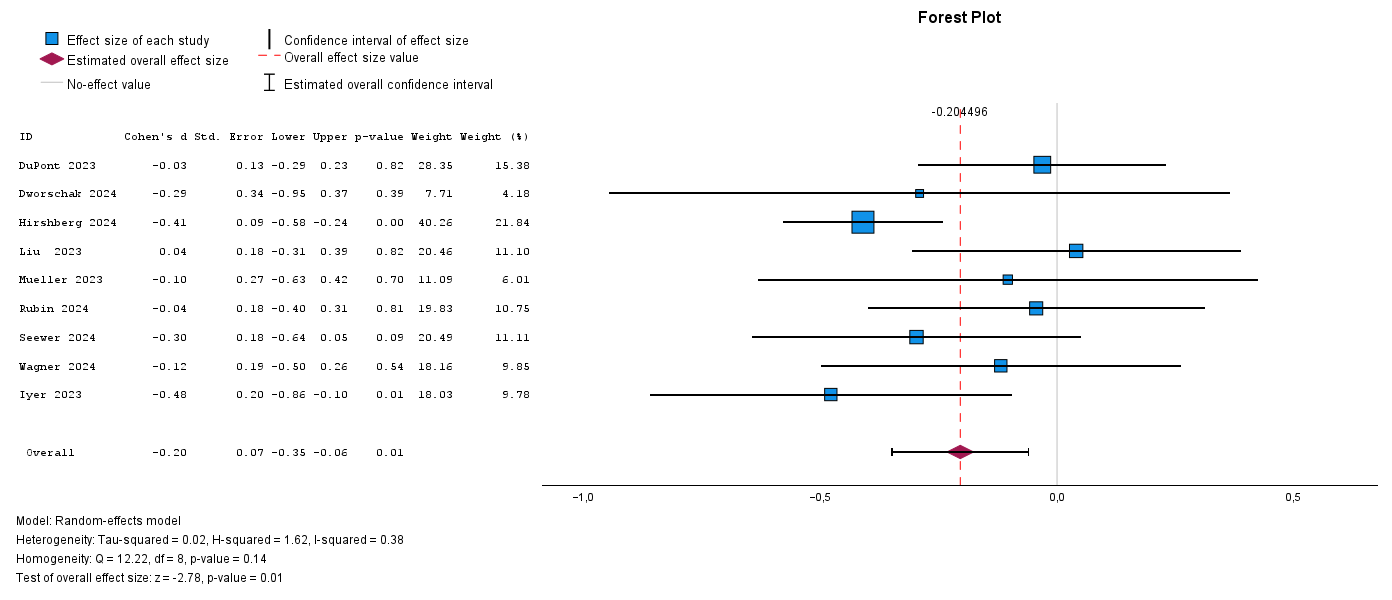


Figure D. Meta-analysis of self-guided psychological interventions on social isolation (2 studies)


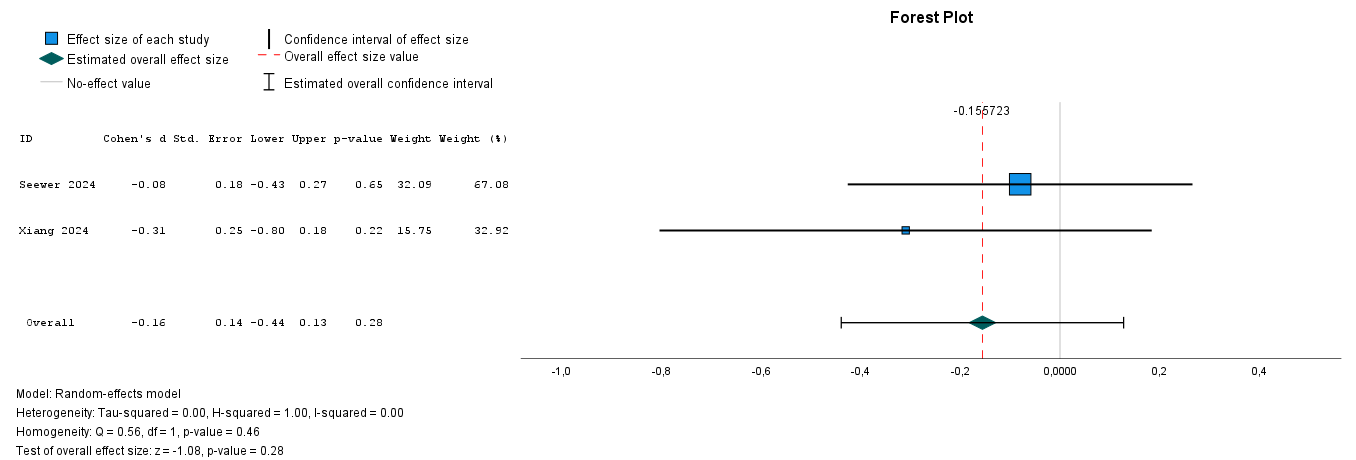


Figure E. Meta-analysis of individual activity on loneliness (2 studies)


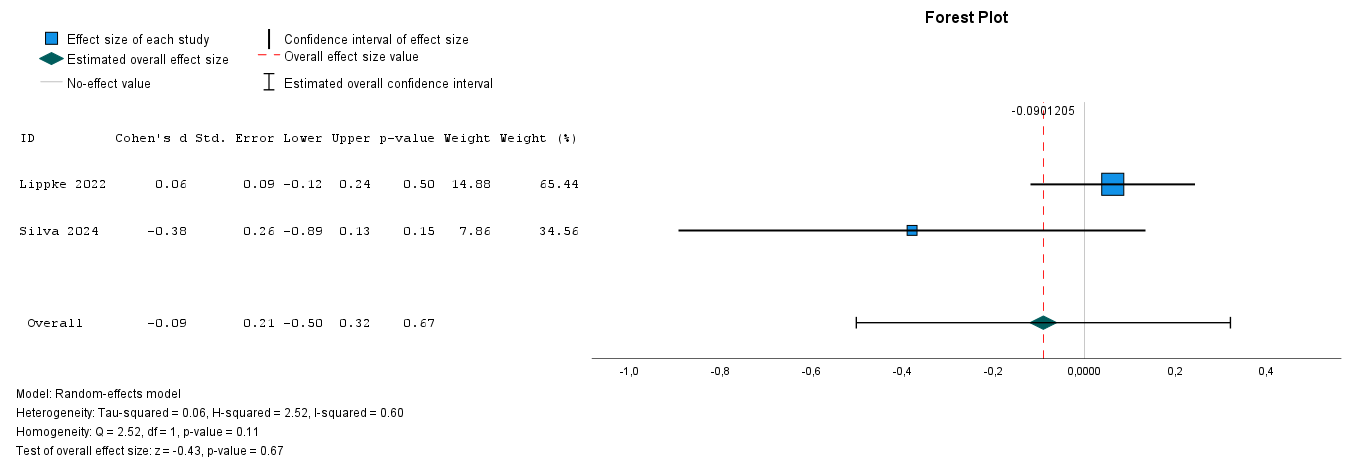


Figure F. Meta-analysis of group activity on social connectedness (2 studies)


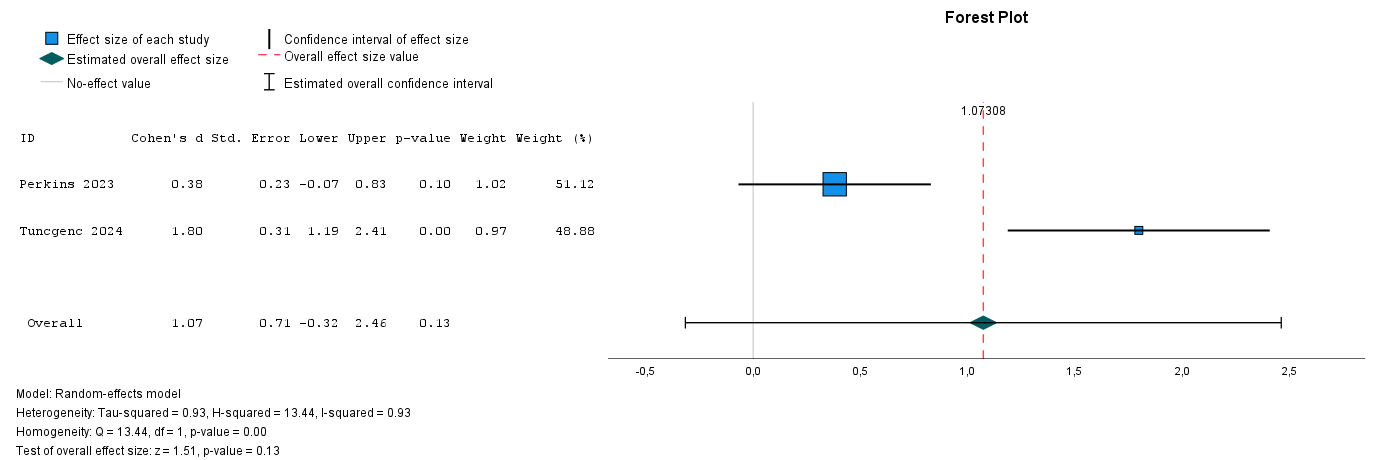


Figure G. Meta-analysis of reduced use of social media on social connectedness (2 studies)


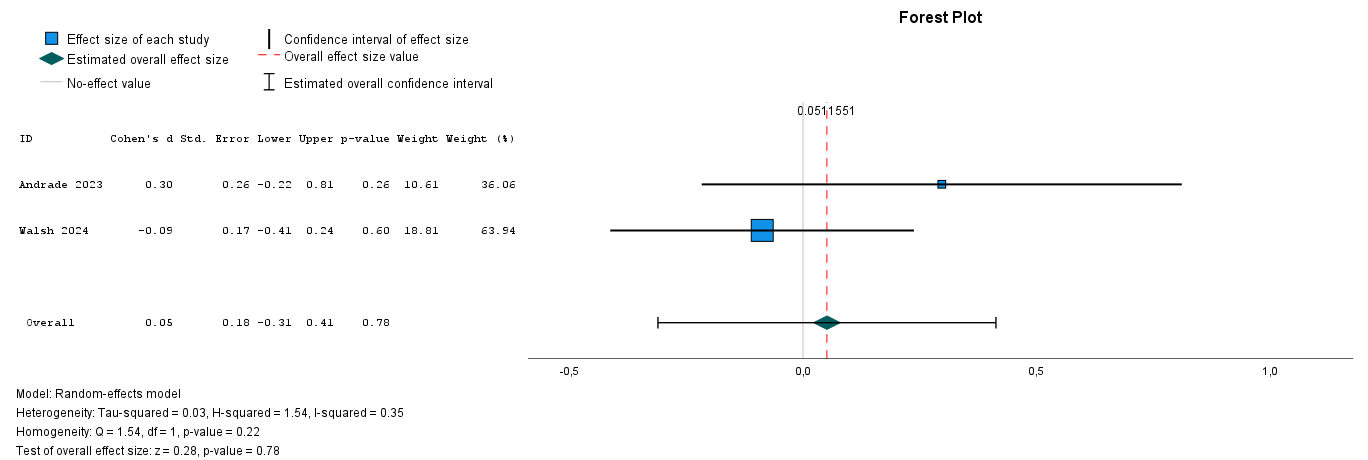


Figure H. Meta-analysis of self-guided psychological interventions on loneliness at follow-up (3 studies)


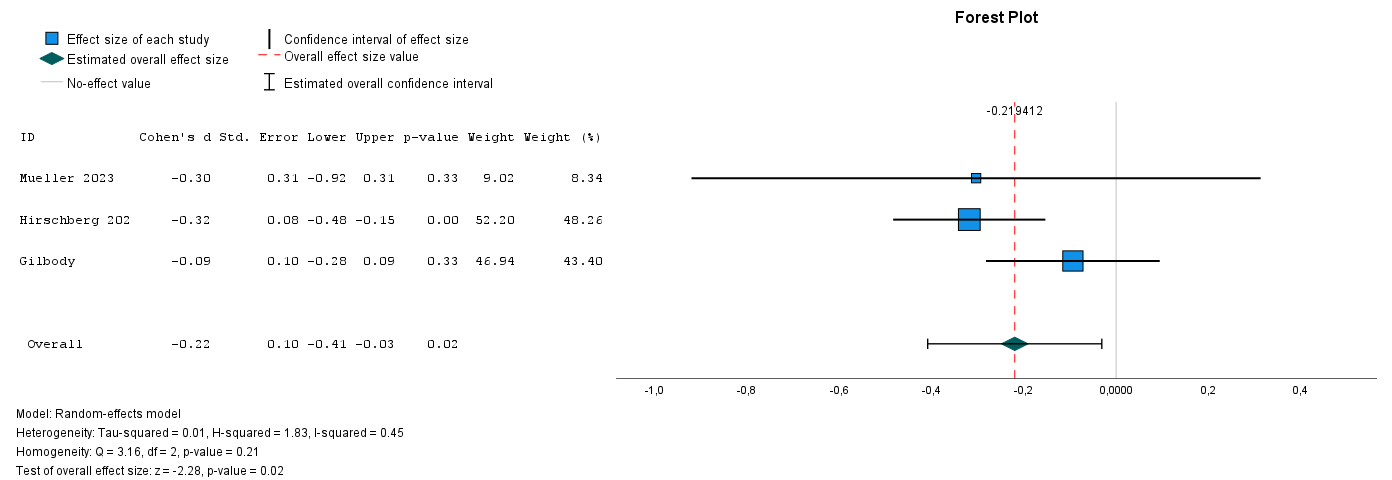


Figure I. Meta-analysis of social support on loneliness (2 studies)


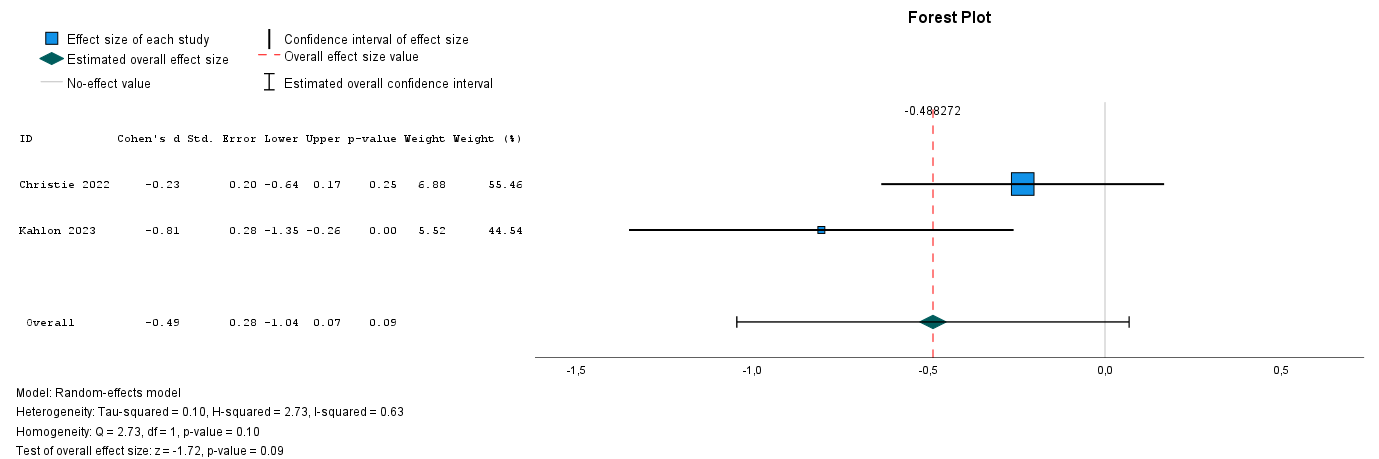


Figure J. Meta-analysis of social support on social isolation (2 studies)


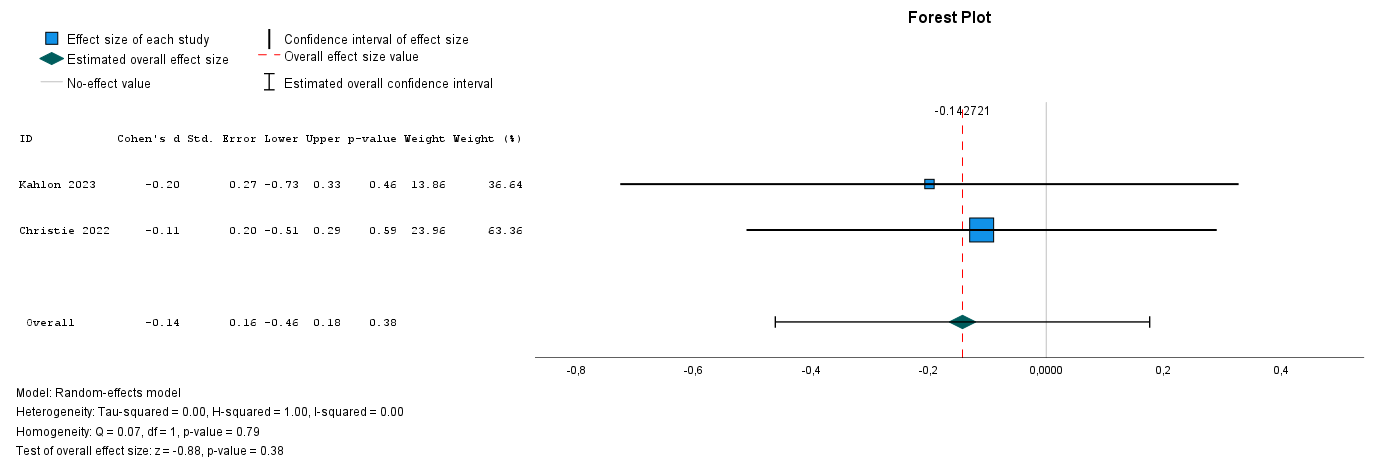


Figure K. Meta-analysis of robotics pets on loneliness (2 studies)


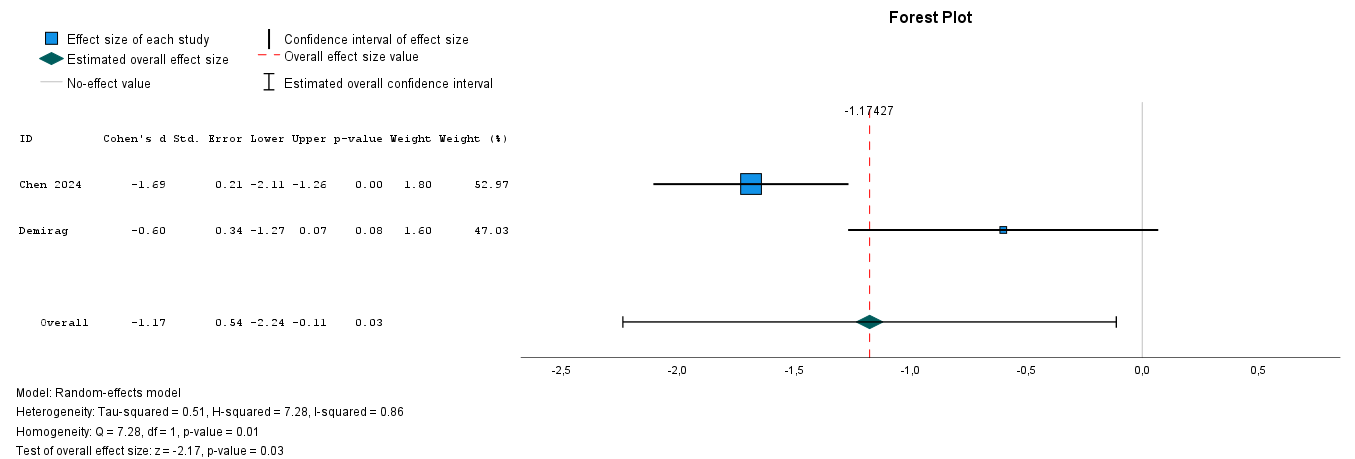


Figure L. Meta-analysis of conversational robots on loneliness (2 studies)


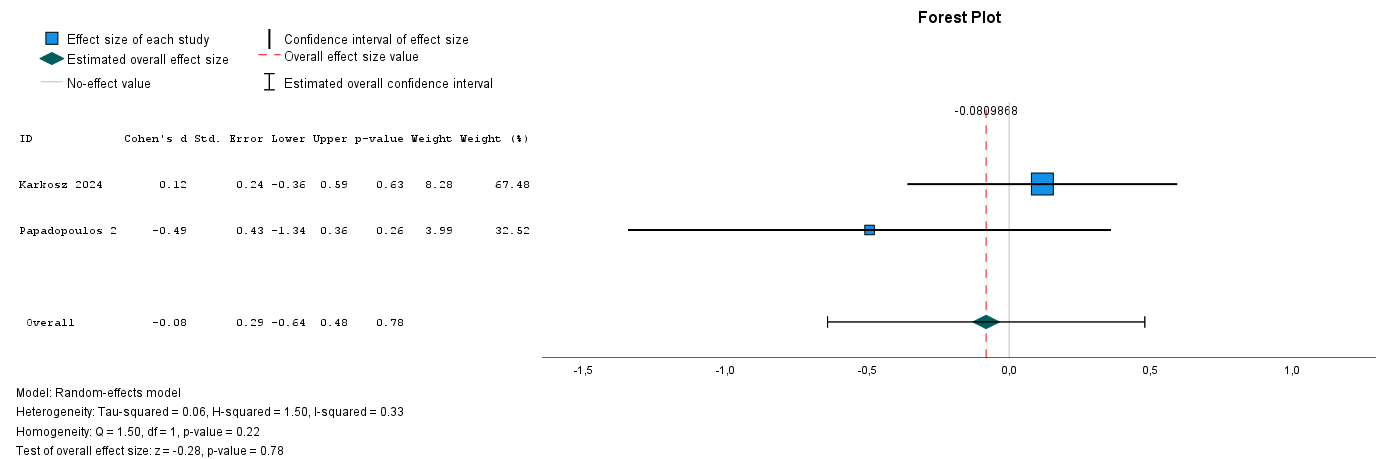


Figure M. Individual studies of various outcomes (5 studies)


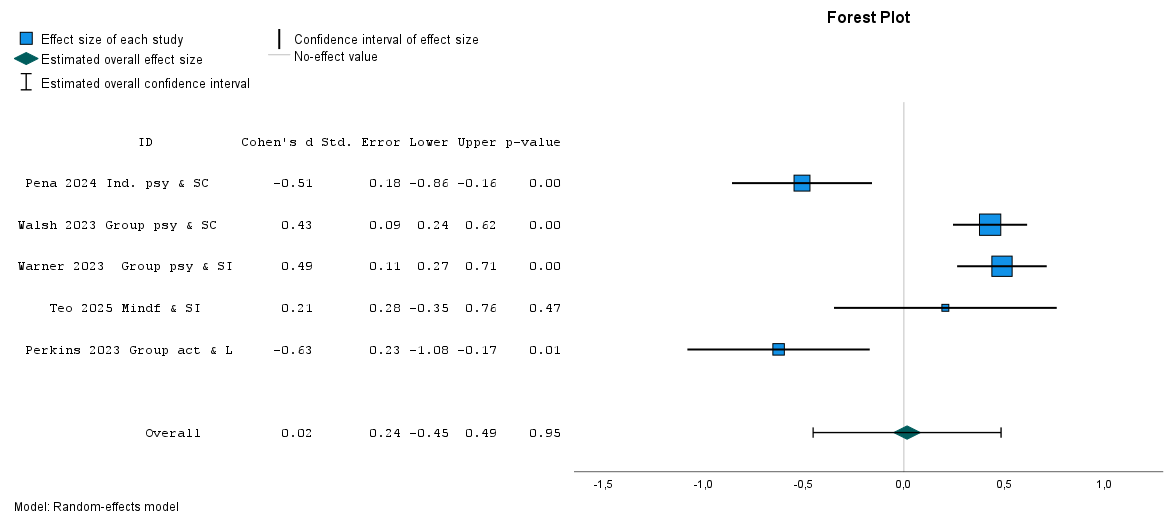


Figure N. Follow up data (11 studies)


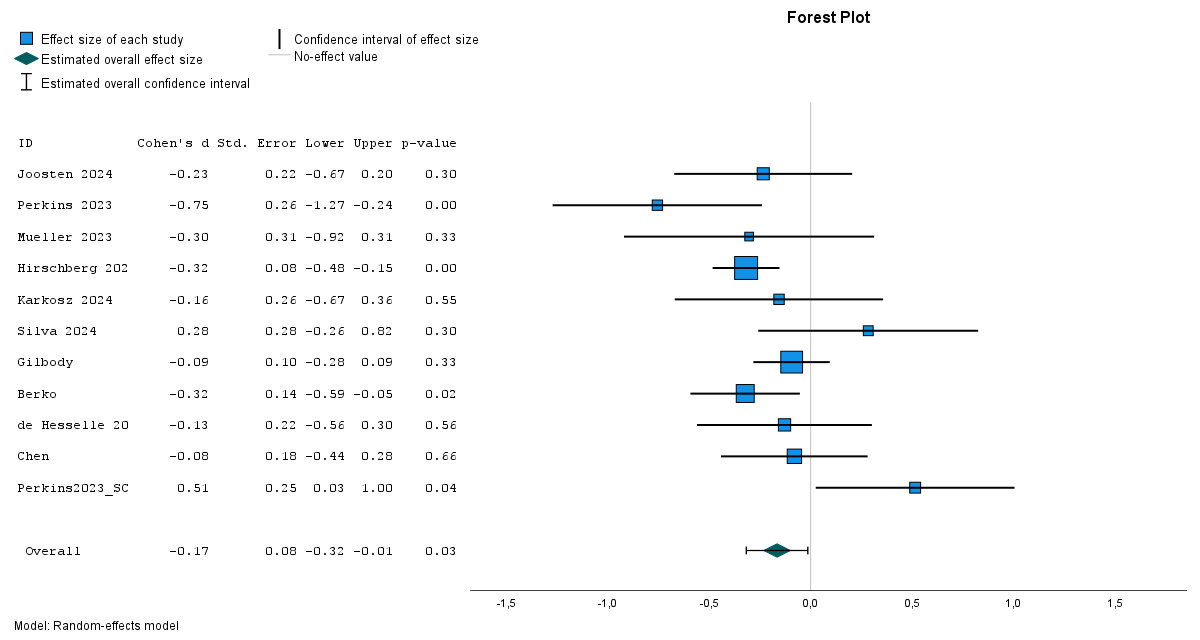


Figure O. Meta-analysis of self-guided psychological interventions on loneliness. Studies (n = 6) with younger participants.


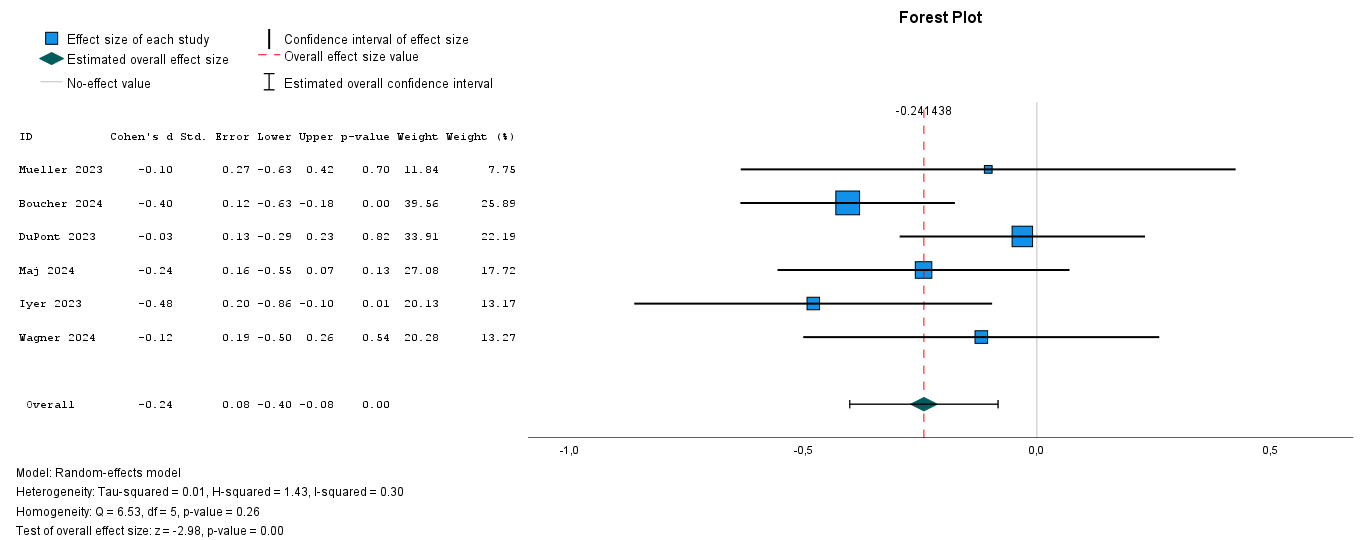


Figure P. Meta-analysis of self-guided psychological interventions on loneliness. Studies (n = 6) with older participants.


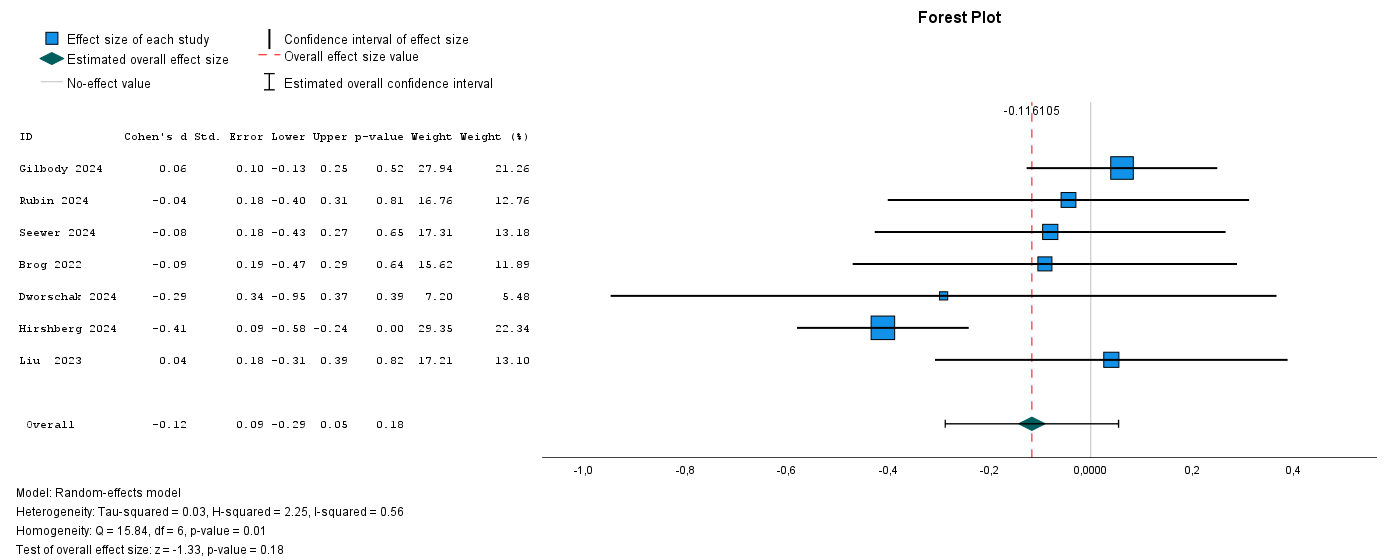


Figure Q. Meta-analysis of group-based psychological interventions on loneliness. Studies (n = 2) with younger participants.


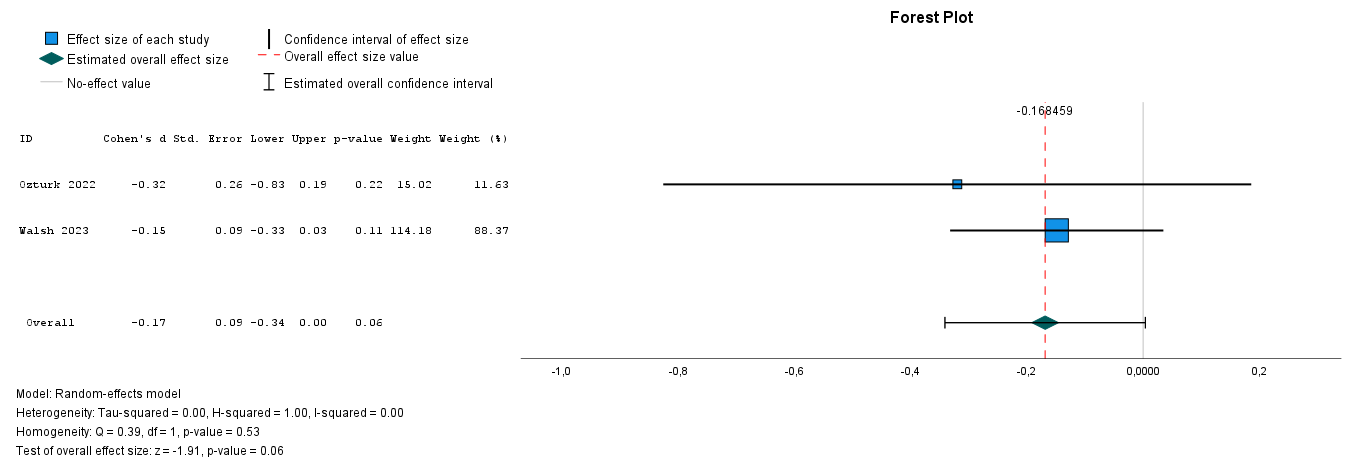


Figure R. Meta-analysis of group-based psychological interventions on loneliness. Studies (n = 4) with older participants.


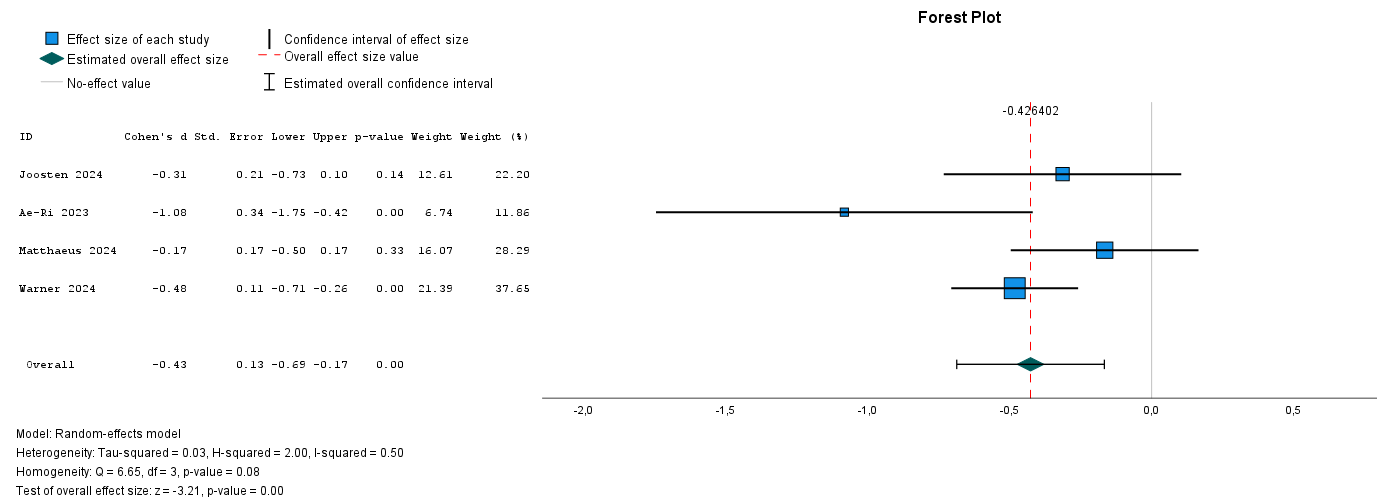

Supplement: Supplementary file 1 — Supplementary material [file mmc1.docx]
